# Smartphone Keyboard Interaction Monitoring as an Unobtrusive Method to Approximate Rest-Activity Patterns: Experience Sampling Study Investigating Interindividual and Metric-Specific Variations

**DOI:** 10.2196/38066

**Published:** 2023-04-07

**Authors:** Karin Smolders, Gerrieke Druijff-van de Woestijne, Kim Meijer, Hannah Mcconchie, Yvonne de Kort

**Affiliations:** 1 Eindhoven University of Technology Human-Technology Interaction group Eindhoven Netherlands; 2 Neurocast Amsterdam Netherlands; 3 King's College London Institute of Psychiatry, Psychology and Neuroscience London United Kingdom

**Keywords:** smartphone keyboard interactions monitoring, rest-activity patters, sleep quality, chronotype, trait self-control, mobile phone

## Abstract

**Background:**

Sleep is an important determinant of individuals’ health and behavior during the wake phase. Novel research methods for field assessments are required to enable the monitoring of sleep over a prolonged period and across a large number of people. The ubiquity of smartphones offers new avenues for detecting rest-activity patterns in everyday life in a noninvasive an inexpensive manner and on a large scale. Recent studies provided evidence for the potential of smartphone interaction monitoring as a novel tracking method to approximate rest-activity patterns based on the timing of smartphone activity and inactivity throughout the 24-hour day. These findings require further replication and more detailed insights into interindividual variations in the associations and deviations with commonly used metrics for monitoring rest-activity patterns in everyday life.

**Objective:**

This study aimed to replicate and expand on earlier findings regarding the associations and deviations between smartphone keyboard–derived and self-reported estimates of the timing of the onset of the rest and active periods and the duration of the rest period. Moreover, we aimed to quantify interindividual variations in the associations and time differences between the 2 assessment modalities and to investigate to what extent general sleep quality, chronotype, and trait self-control moderate these associations and deviations.

**Methods:**

Students were recruited to participate in a 7-day experience sampling study with parallel smartphone keyboard interaction monitoring. Multilevel modeling was used to analyze the data.

**Results:**

In total, 157 students participated in the study, with an overall response rate of 88.9% for the diaries. The results revealed moderate to strong relationships between the keyboard-derived and self-reported estimates, with stronger associations for the timing-related estimates (β ranging from .61 to .78) than for the duration-related estimates (β=.51 and β=.52). The relational strength between the time-related estimates was lower, but did not substantially differ for the duration-related estimates, among students experiencing more disturbances in their general sleep quality. Time differences between the keyboard-derived and self-reported estimates were, on average, small (<0.5 hours); however, large discrepancies were also registered for quite some nights. The time differences between the 2 assessment modalities were larger for both timing-related and rest duration–related estimates among students who reported more disturbances in their general sleep quality. Chronotype and trait self-control did not significantly moderate the associations and deviations between the 2 assessment modalities.

**Conclusions:**

We replicated the positive potential of smartphone keyboard interaction monitoring for estimating rest-activity patterns among populations of regular smartphone users. Chronotype and trait self-control did not significantly influence the metrics’ accuracy, whereas general sleep quality did: the behavioral proxies obtained from smartphone interactions appeared to be less powerful among students who experienced lower general sleep quality. The generalization and underlying process of these findings require further investigation.

## Introduction

### Background

Sufficient sleep is crucial for individuals’ functioning during the wake phase, and sleep disturbances may hamper health and performance [1-7]. The timing and duration of sleep are important determinants of individuals’ affective state and behavior during the wake phase [3,8-11]. In fact, sleep deprivation and irregularity in sleep timing have been associated with a lower sleep quality [9,11-13] and reduced daytime functioning, which is reflected in, for example, sleepiness, fatigue, a lack of vitality, and a more negative mood [11,14-16]. In turn, this can challenge the resistance to engage in unhealthy behaviors and impede a healthy lifestyle [17-19]. Sleep disturbances are prevalent in clinical populations but are also quite common in nonclinical populations [20-22] and may have increased because of the COVID-19 pandemic [23]. Methods for unobtrusively tracking such time-related sleep features (timing and dosage) over longer periods may, therefore, contribute significantly to both scientific health research and practical health applications.

A common method to quantify rest-activity patterns in the field is actimetry, which is obtained with wearable devices that monitor gross motor activity at the wrist. Alternatively, diaries are often used to capture a person’s recalled sleep-wake timing and duration. Despite their merits, these methods require that users wear sensors continuously or frequently respond to questionnaires. Thus, monitoring and quantifying rest-activity patterns over a prolonged period across numerous individuals presents obvious challenges. Although survey data on sleep and wake have been obtained from large samples in cross-sectional studies, these have generally included aggregated, mean-level data, lacking fine-grained information about the temporal dynamics in individuals’ rest-activity patterns. Large-scale and prolonged monitoring of trajectories of sleep parameters in real life could facilitate the use of advanced (time series) data analytic approaches and mathematical modeling of natural rest-activity patterns. This could provide detailed insights into individuals’ habitual rest-activity patterns and regularities or irregularities in these patterns across days, weeks, or years [24-26], potentially elucidating the effects of interventions or treatment [27,28]. Moreover, obtaining and modeling intensive longitudinal data could provide insights into interindividual differences in rest-activity patterns and time-lagged responses to perturbations in sleep. These insights can, in turn, inform the design of adaptive, personalized strategies and just-in-time interventions for promoting healthy sleep-wake patterns. However, this would require unobtrusive, inexpensive, and user-friendly tracking methods.

The widespread use of smartphones offers new opportunities to detect and predict behavioral manifestations related to rest-activity patterns in everyday situations in a noninvasive manner and on a large scale. Recent studies have shown that smartphone-derived metrics can serve as proxies for the timing of sleep and wake by detecting periods of activity and inactivity during the 24-hour day [29-31]. These studies reported strong correlations between keyboard or touchscreen interactions and estimates of rest-activity timing based on diaries or actigraphy but also revealed informative deviations between these different assessment methods. Consistency in the use of smartphones before, during, and after sleep across days may vary from person to person [30]; and interindividual differences were reported in the associations and deviations between smartphone-derived and self-reported [30,31] or physical activity–based putative estimates of the rest and active periods [29,31]. These studies revealed that the agreement between the different assessment modalities was moderated by age, gender, chronotype, and habitual (perisleep) smartphone use [29,30]. Moreover, deviations suggested dissimilar profiles regarding sleep-wake patterns and smartphone use surrounding sleep as a function of the user’s age, employment status (student or university staff), and habitual smartphone use [31].

In addition to chronotype, both trait self-control and general sleep quality have been shown to be determinants—or correlates—of sleep timing and duration as well as screen technology use in the late evening [18,32-40]. Trait self-control has been related to increased (bedtime) procrastination and, via procrastination, to more disturbances in general sleep quality among young adults [36,38]. Lower general sleep quality was associated with shorter sleep duration, higher day-to-day variability in sleep duration, more fragmented sleep, and a larger sleep onset latency, as assessed by actigraphy [16]. There are also indications that persons with lower trait self-control or general sleep quality have a greater tendency to display poor sleep hygiene–related behaviors [41,42], including media technology use surrounding sleep [40,43-45]. However, the extent to which these trait variables related to sleep and sleep habits moderate the associations and deviations between smartphone-based and commonly used metrics for monitoring rest-activity patterns over time is largely unknown.

### Goal of This Study

This study aimed to further investigate the extent to which smartphone interactions can be used to track and quantify rest-activity patterns in situ. To this end, we replicated and expanded on an earlier study on the relationships between smartphone keyboard–derived and self-reported estimates of the onset of rest and active periods and the duration of the rest period during the 24-hour day among students [30]. Complementary to earlier research findings, we aimed to quantify interindividual variations in the relations and deviations between these 2 assessment modalities and assess the extent to which these variations are related to students’ chronotype, general sleep quality, and trait self-control.

## Methods

### Design

This field study used an experience sampling methodology to monitor rest-activity patterns among students by means of user reports, which was combined with continuous smartphone keyboard activity logging. The primary study parameters pertain to the onset of the rest and active periods as well as the duration of the rest period. The study is correlational in nature, and monitoring occurred during the students’ daily routine.

### Recruitment

First-year students registered for a specific course at the Eindhoven University of Technology were recruited. They were informed about the study via a short, recorded message and given the opportunity to ask questions. Students participated in the study as part of a course assignment. They were given the opportunity to receive an alternative assignment if they preferred not to participate.

### Ethics Approval

The recruited students could indicate whether they provided their consent to use their data for research purposes, in addition to educational purposes. In the current analyses, we only included the data of students who provided their consent to participate in the study for research purposes. The study was approved by the Ethics committee of the Human-Technology Interaction group at the Eindhoven University of Technology (ID: 1756).

### Measures

The measures consisted of daily sleep diaries, continuous keyboard activity logging on the students’ smartphones, and 1-time web-based questionnaires. In addition, the participants reported their affective state during the wake episode using experience sampling questionnaires.

#### Sleep Diary

The core Consensus Sleep Diary (CSD) [46] was used to probe self-reported sleep timing, duration, and quality. This core version of the CSD was supplemented with items regarding the consumption of caffeinated and alcoholic drinks from the extended version of the CSD. Moreover, the participants indicated whether they woke up by an alarm, reported their experienced level of fatigue on the previous day, and estimated the number of minutes spent outdoors in daylight on the previous day.

Bedtime, try-to-sleep time, sleep onset (try-to-sleep time + sleep latency), midsleep time, sleep offset, and out-of-bed time were used as estimates of the timing of students’ rest-activity patterns. Moreover, the total bed period (time difference between bedtime and out-of-bed time, in hours) and total sleep period (time difference between sleep onset and sleep offset, in hours) were computed and used as markers for the duration of the rest period.

#### Keyboard Interactions Monitoring

Keyboard activity on the students’ smartphones was continuously and unobtrusively logged using the Neurokeys app (Neurocast BV). The app replaced the native keyboard with a smart keyboard that allowed the registration of keyboard interactions in users’ daily routine without collecting data about the specific letters or numbers typed (to guarantee privacy). Keyboard-derived estimates used in the analyses include the timing of the last keystroke before and the first keystroke after the longest time interval without keyboard activity between 7 PM and 3 PM on the next day, the length of this keystroke-absence period (KAP), and the timing of the midpoint between the last keystroke and first keystroke (midpoint KAP). In addition to these primary parameters, the number of hours during which keyboard activity was detected in the time interval between the first and last keystrokes was computed and included as a potential moderating variable.

#### One-Time Web-Based Questionnaires

Web-based questionnaires were administered to measure trait self-control (short, 13-item, Trait Self-Control Scale [TSC]; [47]), general sleep quality during the past month (Pittsburg Sleep Quality Index [PSQI]; [48]), and chronotype and social jetlag (Ultrashort Munich Chronotype Questionnaire [µMCTQ]; [49]) at the start of the sampling week. Moreover, the level of fatigue experienced during the past week was assessed at the end of the sampling week using the Fatigue Severity Scale [50] (Dutch version [51]).

#### Experience Sampling Questionnaires

The experience sampling questionnaires contained questions regarding momentary sleepiness (Karolinska Sleepiness Scale [52]); vitality, tension, and valence (6 items adopted from Zhang et al [53]); and state self-control (8 items adopted from the State Self-control Capacity Scale [54], similar to Zhang et al [53]). These variables were not analyzed in this study.

### Procedure

After providing informed consent, the participants installed the Neurokeys app and started using the smart keyboard 1 to 3 days before the start of the sampling week to familiarize themselves with the keyboard. In addition, they were asked to complete web-based questionnaires, including the TSC, PSQI, and µMCTQ, and report their general demographics. During the sampling week, the participants continued using the keyboard and received notifications to complete the sleep diary questions each morning at 10:30 for 7 consecutive days. Moreover, the students received notifications to complete the short experience sampling questionnaires at 8 semirandom moments during the wake episode, with at least 30 minutes in between notifications. On the first sampling day, the participants were also reminded to complete the web-based questionnaires regarding trait self-control, general sleep quality, and chronotype and social jetlag. At the end of the sampling week, the participants were asked to complete the FFS. The participants were acknowledged for their participation and received a personalized feedback report a few weeks after the last sampling day. The data were collected in September 2020 in the Netherlands, when mild COVID-19–related restrictions were in force.

### Statistical Analysis

#### Overview

All statistical analyses were performed in R (version 4.1.0, R Foundation for Statistical Computing), using R Studio (R Studio Inc). First, obvious mistakes in the sleep diary data were corrected, and ambiguous values were coded as missing. Next, the distributions and descriptive statistics of the variables of interest were inspected, and outliers (defined as values >4 SD from the mean) were coded as missing. All variables were checked for normality using the Shapiro-Wilk test, and the skewness and kurtosis values were inspected. Owing to the nested structure of the data, multilevel analyses were performed. To account for repeated measures within participants, participant ID was added as a random intercept in all multilevel models (MLMs). An α criterium of .01 was used for all the models to account for multiplicities. *P* values >.01 but <.05 were inspected and classified as nonsignificant trends.

MLMs were first defined to investigate the variance explained at the participant level (between participants) and day level (within participants) in the keyboard-derived and self-reported estimates. These metrics include estimates for the timing of the rest period onset (timing of the last keystroke and self-reported bedtime, try-to-sleep time, and sleep onset), the timing of the midpoint of the rest period (midpoint KAP and self-reported midsleep time), the timing of the activity period onset (timing of the first keystroke and self-reported sleep offset and out-of-bed time), and the duration of the rest period (KAP and self-reported total sleep period and total bed period). To determine the intraclass correlations (ICCs) for each keyboard-derived and self-reported estimate of the students’ rest-activity patterns, we ran unconditional models (null models or intercept-only models; separate model per estimate).

#### Agreement and Differences Between the Keyboard-Derived and Self-reported Estimates

MLMs were then run to investigate whether the differences between the keyboard-derived and self-reported estimates were significantly different from zero. In these models, the values of the self-reported and corresponding keyboard-derived estimates were included as outcome parameter, and modality (keyboard-derived vs self-reported) was added as a fixed factor. The same MLMs were also used to test for equivalence with equivalence bounds set at 0.5 hour as well as at 1 hour. To test for equivalence, the two one-sided tests’ procedure [55] was used. Moreover, Bland-Altman plots, adjusted for repeated measures, were made to display the agreement and differences between the keyboard-derived and self-reported estimates as a function of the mean timing or duration of both assessment modalities. These plots are presented in Multimedia Appendix 1.

Next, we investigated the strength of the association between the keyboard-derived and corresponding self-reported estimates. To this end, a series of MLMs were run with the different self-reported estimates as outcome parameters regressed on the corresponding keyboard-derived metric (separate analysis per outcome parameter). The MLMs testing the associations between the estimates derived via keyboard interactions and those derived via self-reports were subsequently extended with first-level (ie, day-level) predictors, which were expected to interact with the keyboard-derived estimates [30] and comprised the number of hours during which keyboard activity was detected and type of day (weekday vs weekend day). In these models, the keystroke-related metrics were cluster mean centered to facilitate the interpretation of the interaction terms and to distinguish between within-person and between-person slopes. The cluster means (ie, participants’ means) for the last keystroke, first keystroke, midpoint KAP, KAP, and (previous day’s) number of hours with keyboard activity were determined using the random intercepts derived from the unconditional MLMs for the corresponding keyboard-derived estimate. The participants’ mean scores for the last keystroke, first keystroke, midpoint KAP, and KAP were, in addition to the centered scores (ie, daily difference scores: observations − participant mean), added as covariates to each MLM testing moderation by the first-level predictors.

#### Interindividual Variations in the Relational Strength and the Deviations Between Keyboard-Derived and Self-reported Estimates

Random slope MLMs with keyboard-derived estimates (participants’ mean and centered scores) as predictors and the corresponding self-reported estimates as outcome parameters were run to inspect interindividual variation in the relational strength between the centered keyboard-derived estimates and the corresponding self-reported estimates. Next, chronotype, general sleep quality, and trait self-control were added as second-level (ie, participant-level) predictors to these random slope models, including both the “main effects” and the interaction terms with the centered keystroke-derived predictor. These covariates were also grand-mean centered to facilitate the interpretation of the interaction terms. Pearson correlations between these second-level predictors and the variance inflation factors based on the MLMs were first inspected to check for potential issues related to multicollinearity.

In addition to these assessments of the associations between the keyboard-derived and self-reported estimates, we also computed the differences between the timing or duration estimated with keyboard interactions and the corresponding sleep diary data and investigated the metric-specific and interindividual variations in the magnitude and direction of these differences. These models and their corresponding findings are described in Multimedia Appendix 1.

## Results

### Sample Statistics

In total, 157 students (n=77, 49% male and n=80, 51% female), with a mean age of 19.38 (SD 1.88; range 17-25) completed the sleep diaries. Native Dutch students (136/157, 86.6%) received the questions in Dutch and the remaining 13.4% (21/157) of students in English. The µMCTQ revealed that the timing of midsleep on work- and lecture-free days (corrected for sleep debt accumulated during work and lecture days; midsleep on free days, corrected for accumulated sleep debt) was 04:50 on average (SD 01:01; range 02:30-07:32). The average (absolute) social jetlag was 1.22 hours (SD 0.73; range 0-3.5). On average, the participants showed at least some disturbances in their general sleep quality (mean 5.16, SD 2.27; range 1-13), as assessed using the PSQI. Scores on the TSC scale ranged from 1.31 to 4.31, with an average of 2.98 (SD 0.56).

The overall response rate for the diaries was 88.9%. As shown in Figure S1 in Multimedia Appendix 1, most students (105/157, 66.9%) completed all diaries. The number of participants who completed the diary dropped slightly toward the end of the sampling week, with a minimum of 76.4% (120/157) of participants completing diaries on Sunday (the 7th sampling day). On 60% of the weekdays (Monday to Friday), the participants were woken up by an alarm, compared with 46% of the weekend days. In total, 2,757,199 keystroke events were generated from September 14 to 20, 2020. On average, keyboard activity was detected during 12.3 hours per day (SD 4.0; range 1-21), with the keyboard used for more hours on weekdays (mean 12.7, SD 3.9) than on weekends (mean 11.0, SD 4.1).

### Descriptive Statistics for Rest-Activity Estimates

Table 1 shows the corresponding descriptive statistics for the various rest-activity estimates, including the ICCs. The ICCs revealed that most of the variance in the estimates (onset of rest period, midpoint of the rest period, onset of active period, and duration of the rest period) can be explained at the first level (ie, day level). For the timing-related estimates, 21%-30% of the variance can be explained at the second level (ie, participant level), with very similar percentages for the keyboard-derived and self-reported estimates. For self-reported estimates, the variance in the duration of the rest period at the participant level was lower than that in timing-related estimates, suggesting lower consistency in the self-reported duration of the rest period across days within participants. By contrast, for keyboard-derived measures, the percentage of variance in KAP at the participant level was higher and similar to the variance in the timing-related estimates (Table 1).

**Table 1:** Descriptive statistics for the keyboard-derived and self-reported estimates of rest-activity timing and duration.

	Mean (SD)	Minimum	Maximum	Skewness	Kurtosis	ICC^a^
Bedtime	23:59:34 (01:33:30)	7 PM	5:45 AM	0.64	3.48	0.23
Try-to-sleep time	00:30:55 (01:28:09)	8:20 AM	6:15 AM	0.65	3.64	0.23
Sleep onset	00:46:47 (01:25:31)	8:30 PM	6:30 AM	0.63	3.71	0.22
Midsleep time	04:39:52 (01:11:54)	1:08 AM	8:58 AM	0.59	3.36	0.29
Sleep offset	08:31:38 (01:19:08)	3:54 AM	12:55 PM	0.34	3.50	0.28
Out-of-bed time	09:01:18 (01:22:13)	5:15 AM	1:50 PM	0.34	3.07	0.21
Total sleep period	07:45:32 (01:20:58)	2:23 AM	12:30 PM	−0.38	3.86	0.11
Total bed period	09:02:46 (01:29:06)	3:31 AM	2:50 PM	−0.18	3.69	0.13
Last keystroke	00:02:06 (01:35:50)	7:27 PM	6:11 AM	0.24	3.43	0.26
Midpoint KAP^b^	04:24:21 (01:14:06)	1:03 AM	9:16 AM	0.52	3.35	0.28
First keystroke	08:46:23 (01:28:46)	3:27 AM	2:10 PM	0.41	3.88	0.30
KAP	08:42:51 (01:52:18)	2:58 AM	3:44 PM	0.36	4.03	0.27

^a^ICC: intraclass correlation (determined using the corresponding unconditional [intercepts-only] model, and represents the variance explained at the participant level).

^b^KAP: keystroke-absence period.

### Quantification of Agreement and Differences Between the Keyboard-Derived and Self-reported Estimates

**Figure 1:**
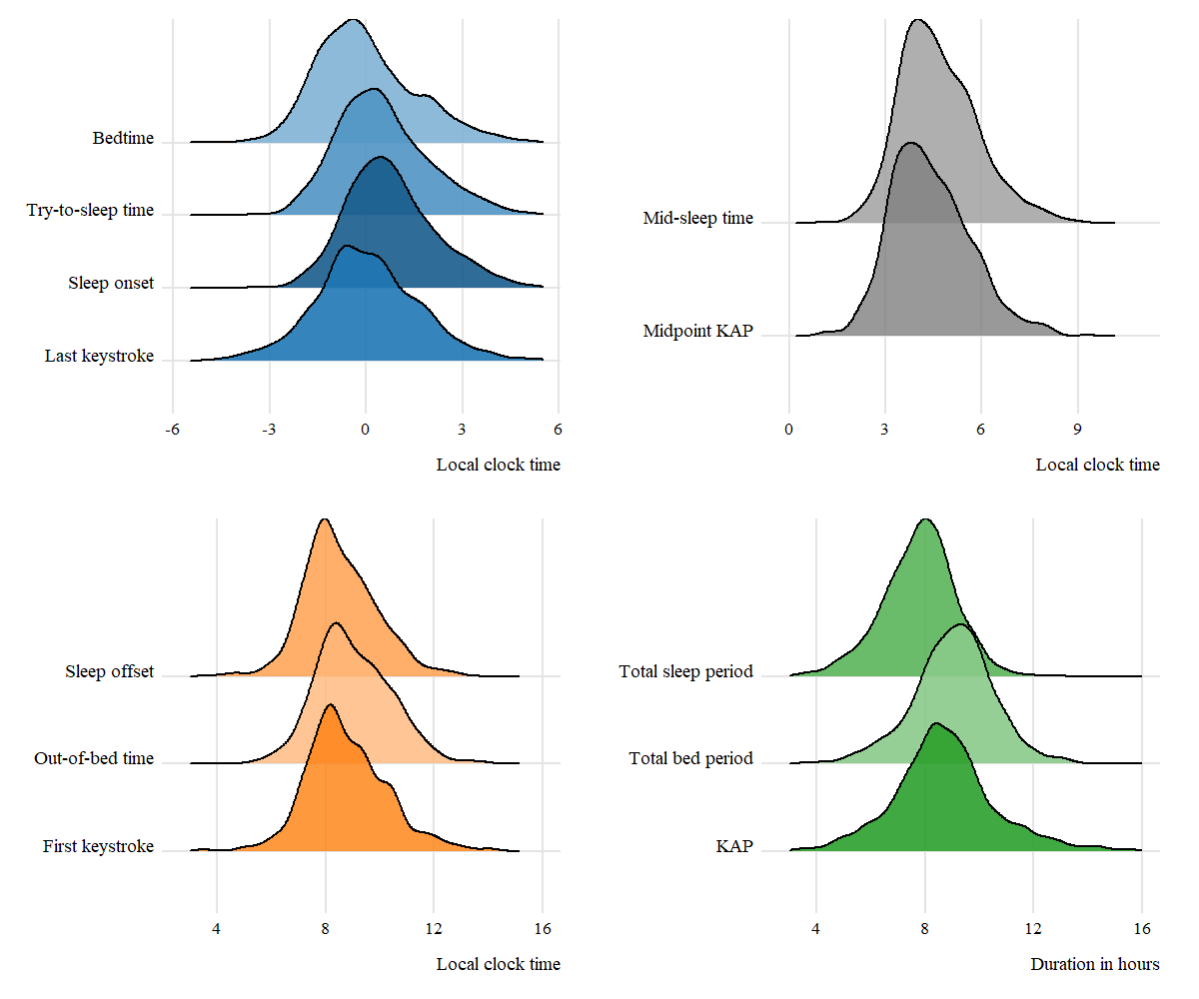
Ridgeline plots of rest-activity pattern estimates derived from the sleep diary and those derived from the keystroke logging data. KAP: keystroke-absence period.

**Figure 2:**
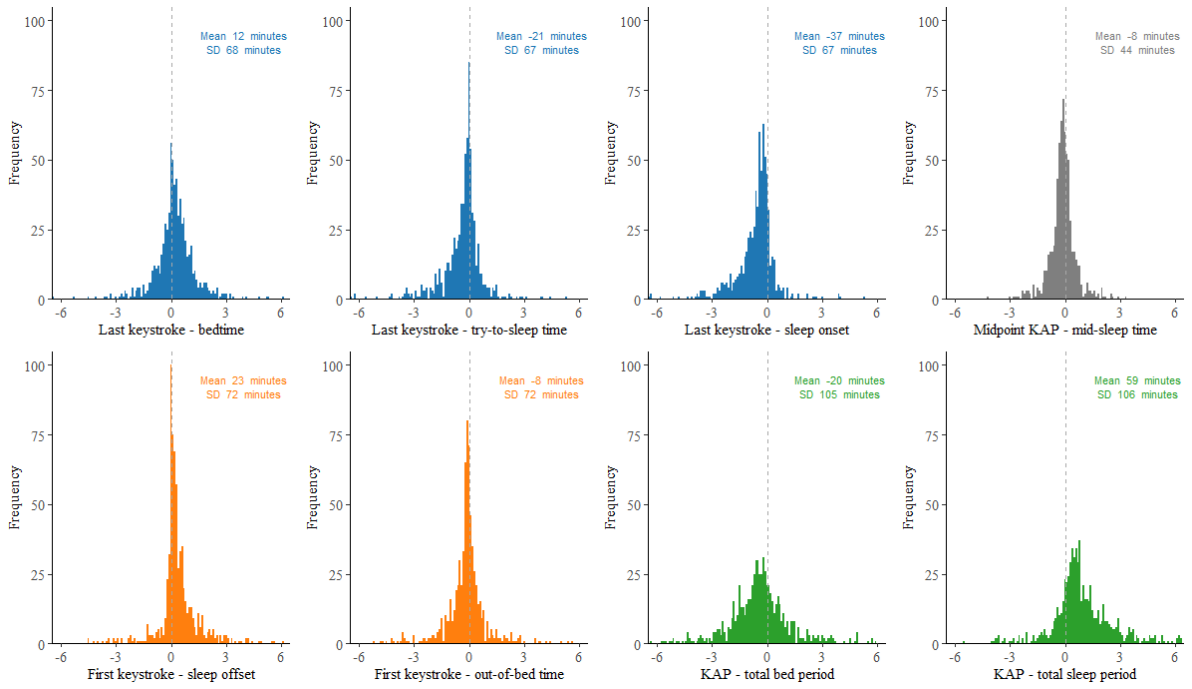
Distributions of the difference scores between keyboard-derived and self-reported estimates (in hours). KAP: keystroke-absence period.

Figure 1 shows the overlap between the distributions of the various estimates of the timing of rest and activity periods and the duration of the rest period assessed with keystroke logging and the sleep diary. Figure 2 displays the calculated differences between the keyboard-derived and self-reported estimates of rest onset, midpoint, offset, and duration (also refer to Table S1 in Multimedia Appendix 1 for the descriptive statistics of the deviations, including the ICCs).

The vertical dashed lines correspond to a difference of zero. Values smaller than zero represent a later timing or longer duration for the self-reported estimates than for keyboard-derived estimates, and positive values reflect an earlier timing or a shorter duration for the self-reported estimates than for the keyboard-derived estimates. Figure S2 in Multimedia Appendix 1 shows the Bland-Altman plots of the rest-activity pattern estimates derived from the sleep diary and keystroke logging data.

**Figure 3:**
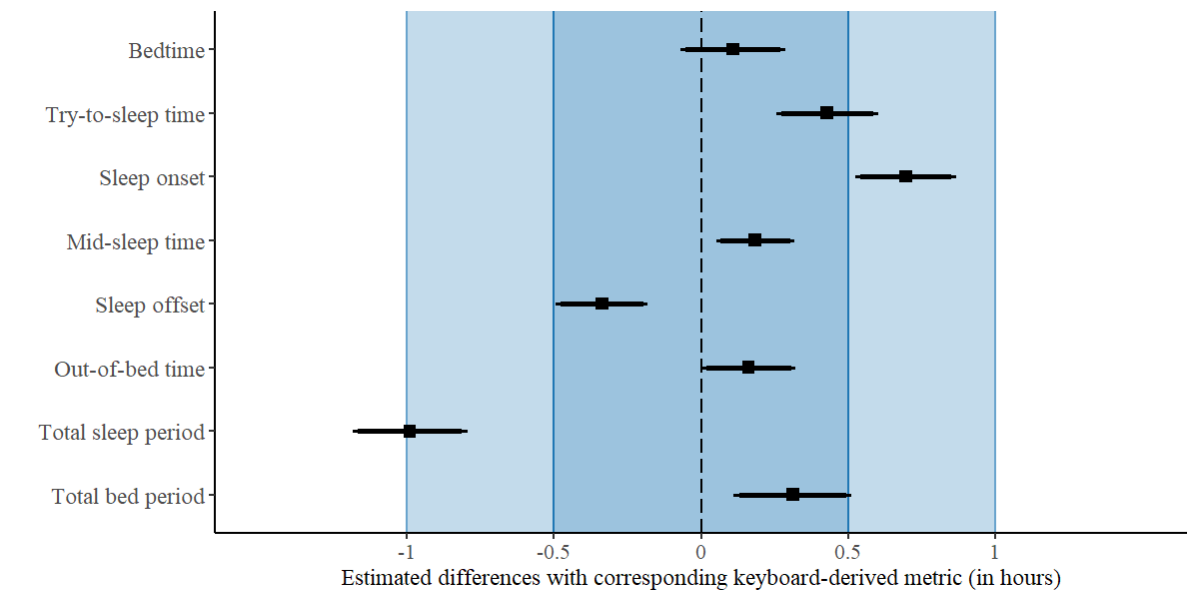
Forest plot for differences between the keyboard-derived and self-reported estimates of rest-activity timing and duration of the rest period.

Differences between the keyboard-derived and the corresponding self-reported estimates were tested for statistical significance and for statistical equivalence (defined as deviations <0.5 hour or deviations <1 hour) using multilevel modeling. The results of the null-hypothesis significance tests revealed that all timing-related and duration-related estimates of the participants’ self-reported rest-activity patterns were statistically different from the corresponding keyboard-derived estimates (all *P*<.001), except for the difference between the self-reported estimate of the timing of bedtime and the timing of the last keystroke, which was not statistically significant (*P*=.12; Figure 3). Equivalence tests against Δ_L_=−0.5 hour and Δ_U_=0.5 hour yielded significant results for the deviations between the last keystroke and self-reported bedtime, between midpoint KAP and self-reported midsleep time, between the first keystroke and self-reported sleep offset and out-of-bed time (all *P*<.001), and between KAP and self-reported total bed period (*P*=.01). This indicated that these differences were not significantly longer than 30 minutes (Figure 3). Equivalence was rejected for the difference between the timing of the last keystroke and self-reported try-to-sleep time (*P*=.14) and sleep onset (*P*>.99) and between KAP and self-reported total sleep period (*P*>.99), indicating that these estimates deviated, on average, by >0.5 hour. All differences were statistically equivalent with equivalence bounds of +1 hour and −1 hour (all *P*<.001), except for the difference between KAP and the total sleep period (*P*=.44; Figure 3).

In Figure 3, the thick bars represent the 98% CIs used for the two one-sided tests’ analyses. The thin bars represent the 99% CIs used for the null-hypothesis significance tests. Negative differences refer to an earlier timing or a shorter duration for the keyboard-derived estimate than for the self-reported estimate.

### Relations Between Keyboard-Derived and Self-reported Estimates of Rest-Activity Patterns

Table 2 shows the results of the MLMs with the self-reported estimates as outcome parameters regressed on the corresponding keyboard-derived estimates. The timing of the last keystroke was significantly related to all 3 self-reported estimates of the rest period onset, with an estimated explained variance (R-squared) of 0.57 to 0.58. The timing of the first keystroke was significantly associated with the self-reported estimates of the onset of the active period, and the midpoint of KAP was significantly related to the self-reported midsleep time. Although the R-squared value for the prediction of self-reported midsleep time by midpoint KAP was somewhat higher than those for the rest onset period estimates (R-squared=0.66), the estimated explained variance was somewhat lower for self-reported timing of sleep offset and out-of-bed time regressed on the timing of the first keystroke (R-squared=0.41 for both estimates). KAP was also significantly related to the self-reported estimates of the duration of the rest period, but the predictions of the total sleep period and total bed period yielded the lowest R-squared values, suggesting that only 26%-27% of the variance in self-reported duration of the rest period could be explained by KAP.

**Table 2:** Results of the multilevel models with keyboard-derived estimates as predictor for self-reported estimates of rest-activity timing and duration.

Keyboard-derived predictor and self-reported outcome		β	0.5%	99.5%	*t* test (*df*)	*P* value
**Last keystroke**						
	Bedtime	.74	0.67	0.81	28.67 (698.9)	<.001
	Try-to-sleep time	.74	0.68	0.81	28.70 (699.2)	<.001
	Sleep onset	.75	0.68	0.81	28.93 (699.0)	<.001
**Midpoint KAP^a^**						
	Midsleep time	.78	0.72	0.84	34.30 (678.7)	<.001
**First keystroke**						
	Sleep offset	.61	0.53	0.68	20.48 (698.3)	<.001
	Out-of-bed time	.63	0.55	0.70	20.70 (677.7)	<.001
**KAP**						
	Total sleep period	.51	0.42	0.60	14.58 (682.7)	<.001
	Total bed period	.52	0.43	0.60	15.09 (681.4)	<.001

^a^KAP: keystroke-absence period.

### First-Level Moderators: Hours With Keyboard Activity and Type of Day

The results revealed that the relationships between the keyboard-derived and self-reported estimates were moderated by the (previous) number of hours during which keyboard activity was present for all the estimates of the timing of the rest period onset and the active period onset and the duration of the rest period (all *P*<.001; Tables S2-S9 in Multimedia Appendix 1). The associations between the timing of the last keystroke and self-reported bedtime, try-to-sleep time, and sleep onset were stronger when participants had had more hours with keyboard activity on the previous day was larger than their week average (Tables S2-S4 in Multimedia Appendix 1). Similarly, the relational strength between the timing of the first keystroke and self-reported sleep offset and out-of-bed time was higher when the participants used the keyboard during relatively more hours on the next day (Tables S5 and S6 in Multimedia Appendix 1). For self-reported midsleep time, total bed period, and total sleep period, the interaction term with the number of hours with keystrokes on the next day, but not on the previous day, was significant, suggesting stronger associations between the keyboard-derived and self-reported estimates when participants used the keyboard during more hours on the following day than their week average (Tables S7-S9 in Multimedia Appendix 1). However, there was also a nonsignificant trend for stronger associations between KAP and self-reported total sleep period when the participants used the keyboard during more hours on the previous day. The interaction terms with type of day suggested no statistically significant differences in the relational strength between the keyboard-derived and self-reported estimates for the onset of the rest period, onset of the active period, or duration of the rest period (Tables S2-S9 in Multimedia Appendix 1). Similar to the associations between the estimates obtained with the 2 modalities, the regularity of keyboard use was also significantly or near-significantly associated with (most of) the time differences between the keyboard-derived and self-reported estimates, whereas type of day did not predict the magnitude of these difference scores (Multimedia Appendix 1).

Figure S3 in Multimedia Appendix 1 shows boxplots of the rest-activity estimates for weekdays and weekend days separately, and Table S10 in Multimedia Appendix 1 shows the results of MLMs investigating type-of-day moderations in keyboard-derived and self-reported estimates. These findings showed that all timing-related estimates occurred, on average, later on weekend days than on weekdays, whereas there were no statistically significant differences in the rest duration–related estimates as a function of type of day. The differences in the timing-related estimates between weekdays and weekend days were detected for both modalities, albeit slightly more pronounced for the self-reported than for keyboard-derived estimates.

### Interindividual Variation in the Relational Strength Between Keyboard-Derived and Self-reported Estimates

**Figure 4:**
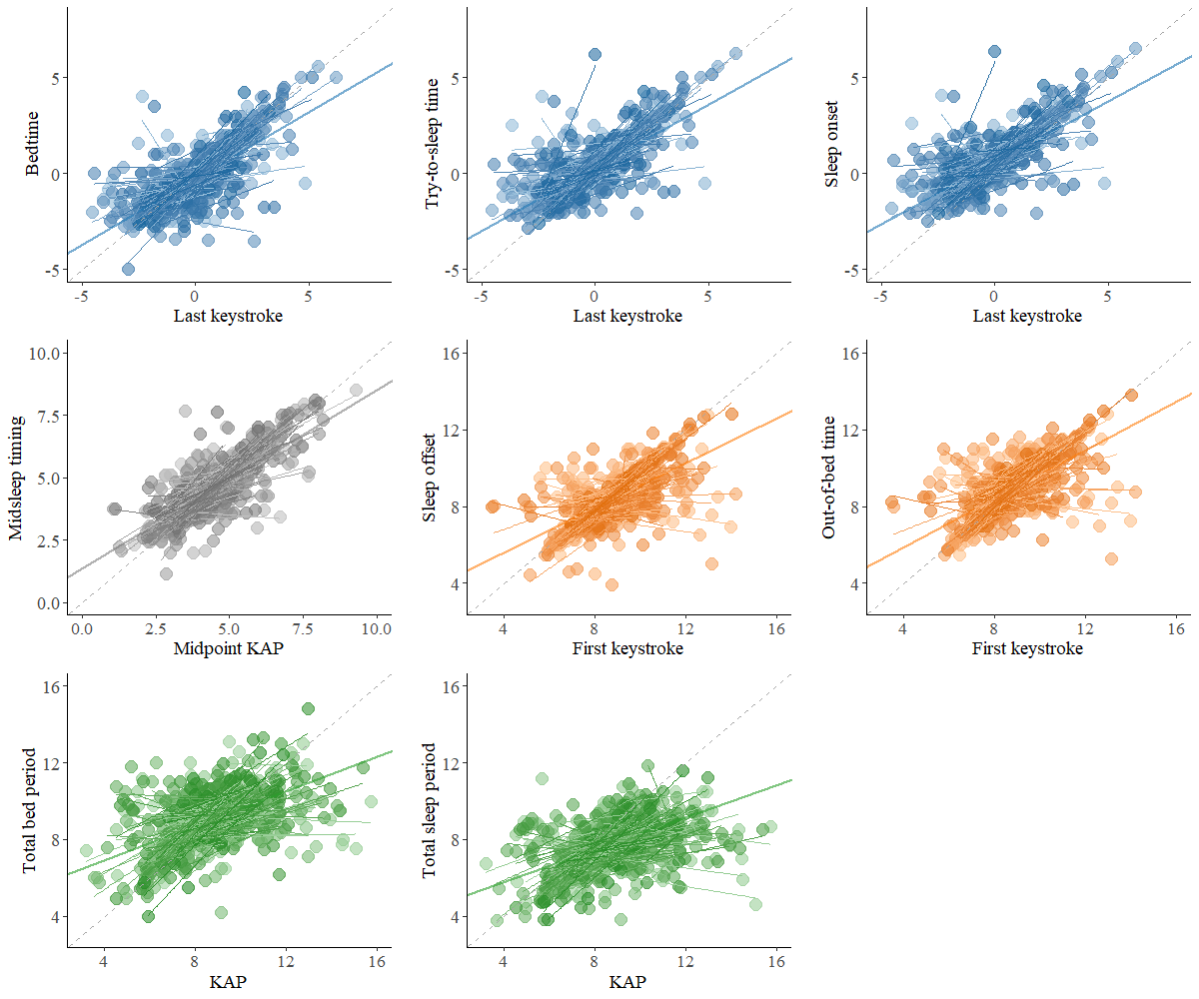
Scatterplots with fixed and random slopes for the associations between the keyboard-derived and self-reported estimates. KAP: keystroke-absence period.

The results of the random slope MLMs with only the keyboard-derived metrics (cluster mean and cluster mean centered scores) as predictors and the corresponding self-reported metrics as outcome parameters are presented in Table 3. Random slopes were statistically significant for all self-reported estimates regressed on the corresponding cluster mean centered keyboard-derived estimate, indicating a slope variation of 0.25 to 0.39 SD. The scatter plots in Figure 4 display the associations between the keyboard-derived and self-reported estimates, including the fixed and random slopes.

In Figure 4, the dashed line represents a perfect relationship. The thicker solid line displays the fixed slope. The thinner lines display the random slopes per participant.

**Table 3:** Statistics of random slope models^a^.

Keyboard-derived predictor and self-reported outcome			SD of slopes across participants		Log likelihood		AIC^b^		LRT^c^ (*df*)		*P* value
**Last keystroke**											
	Bedtime	0.37		−975.16		1960.32		94.56 (2)		<.001	
	Try-to-sleep time	0.35		−931.59		1873.18		97.61 (2)		<.001	
	Sleep onset	0.34		−903.92		1817.83		102.04 (2)		<.001	
**Midpoint KAP^d^**											
	Midsleep time	0.25		−678.07		1366.14		40.53 (2)		<.001	
**First keystroke**											
	Sleep offset	0.36		−934.55		1879.11		117.72 (2)		<.001	
	Out-of-bed time	0.39		−984.03		1978.06		103.12 (2)		<.001	
**KAP**											
	Total sleep period	0.32		−1073.36		2156.73		69.18 (2)		<.001	
	Total bed period	0.32		−1136.72		2283.45		36.87 (2)		<.001	

^a^Keyboard-derived estimates refer to the cluster mean centered scores. Statistics represent the slope variation (displayed in SDs) and the likelihood ratio tests of the multilevel model in which the random slope for the cluster mean centered score is reduced. A significance test indicates that the model becomes significantly better if the random slope for the keyboard-derived metric is included.

^b^AIC: Akaike information criterion.

^c^LRT: likelihood ratio test.

^d^KAP: keystroke-absence period.

### Second-Level Moderators: Chronotype, General Sleep Quality, and Trait Self-control

Cross-level interactions between the keyboard-derived metrics and chronotype, general sleep quality (PSQI score), and trait self-control revealed that only general sleep quality was a significant or near-significant moderator for the relationship between the keyboard-derived and self-reported estimates of the timing of the onset of the rest and active periods and the midpoint of the rest period (Tables S12-S17 in Multimedia Appendix 1). The relational strength between the timing of the last keystroke and self-reported bedtime was lower among students with a higher PSQI score (indicating more disturbances in general sleep quality). The associations between midpoint KAP and midsleep time and between the timing of the first keystroke and self-reported sleep offset were also less strong among students who reported more disturbances in general sleep quality. Similar, but nonsignificant, trends were observed for the predictions of self-reported try-to-sleep time, sleep onset, and out-of-bed time (*P*=.03, *P*=.05, and *P*=.03, respectively). Chronotype and trait self-control did not significantly moderate the predictions of self-reported timing of the onset of the rest and active periods by the corresponding keyboard-derived metrics (see Tables S12-S17 in Multimedia Appendix 1). Inspection of the “main effects” of these second-level predictors revealed that chronotype was a statistically significant predictor of try-to-sleep time, sleep onset, midsleep time, sleep offset, and out-of-bed time, with, as expected, later timings among later chronotypes (see Tables S13-S17 in Multimedia Appendix 1). A similar nonsignificant trend was observed for the association between chronotype and self-reported bedtime (*P*=.04). Trait self-control was not significantly related to any of the timing-related self-reported estimates but showed a nonsignificant trend for an earlier bedtime, try-to-sleep time, and sleep onset among students with a higher trait self-control (*P*=.04, *P*=.01, and *P*=.01, respectively). There was also a significant association between sleep onset and the PSQI score, indicating a later self-reported sleep onset among students experiencing more disturbances in sleep quality (*P*=.01).

The results for the duration-related estimates revealed no statistically significant moderations in the relationship between KAP and the self-reported estimates as a function of chronotype, general sleep quality, and trait self-control (see Tables S18 and S19 in Multimedia Appendix 1). The relationship between KAP and both total bed period and total sleep period did show a nonsignificant trend for a stronger association among later chronotypes (both *P*=.01). The “main effects” of chronotype and general sleep quality on self-reported total sleep period and total bed period were not statistically significant (see Table S18 and Table S19 in Multimedia Appendix 1). Trait self-control was not significantly related to the self-reported total bed period but showed a nonsignificant trend for a longer total sleep period among students with higher trait self-control (*P*=.03).

The results of the MLMs inspecting the time differences between the keyboard-derived and self-reported estimates regressed on chronotype, general sleep quality, and trait self-control showed that general sleep quality was a significant or near-significant predictor of the magnitude, but not the direction, of these differences (Tables S20 and S21 in Multimedia Appendix 1). Chronotype and trait self-control were not significantly associated with the time difference scores between the estimates obtained from keyboard interactions and those obtained from the sleep diary (Table S20 in Multimedia Appendix 1).

## Discussion

### Principal Findings and Comparison With Prior Work

This study replicated earlier research findings [29-31], showing that the timing of the last keystroke and the first keystroke surrounding the nocturnal prolonged keyboard inactivity period on the smartphone can serve as good predictors of the self-reported timing of the rest and active period onsets. These findings also complemented the associations between smartphone interactions and putative sleep-wake timing derived from actimetry [29,31]. Together, these studies support the potential of smartphone keyboard monitoring as an unobtrusive method for obtaining a behavioral proxy for rest-activity patterns.

Despite the substantial agreement between the assessment methods, the results of this study and earlier studies showed that the mapping of the estimates obtained with the different assessment modalities is not perfect. In fact, deviations, reflected in both overestimations and underestimations, rendered informative insights for the monitoring of rest-activity patterns and perhaps even the diagnosis and treatment of disturbances in these patterns.

Inspection of the associations between the various keyboard-derived and self-reported estimates showed metric-specific variation in the agreement between the 2 assessment modalities. First, the associations were slightly stronger for the timing of the onset of the rest period than for the timing of the onset of the active period. Moreover, although the timing-related estimates showed moderate to strong relationships, the associations between KAP and the self-reported total bed period and total sleep period were substantially weaker. In fact, the findings suggested that approximately 75% of the variance in the total bed or sleep period remained unexplained. Earlier studies also revealed lower explained variances in the putative duration of the rest period, with similar percentages [29,30]. A potential explanation for these weaker associations is that the duration-related estimates are based on 2 events surrounding the prolonged inactive period, which are both likely prone to inaccuracies in the approximation. Nevertheless, although the estimates for the midpoint of the rest period were also based on both events, the strength of the association between midpoint KAP and midsleep time was similar to the strength of the associations for the rest onset period. This might be explained by the fact that potential inaccuracies in the estimates for both events are added in the computation of the duration-related metrics but divided to determine the timing of the midpoint of the rest period.

The differences in the strength of the associations between the timing-related and duration-related estimates were not reflected in the deviations between the keyboard-derived and self-reported estimates. In fact, the metric-specific variations in the deviations suggested smaller discrepancies between the bed-related versus the sleep-related estimates. Similar to the findings of Druijff-van de Woestijne et al [30], the keyboard-derived estimates of the timing of the onset of the rest and active periods and the duration of the rest period were, on average, closer to the bed-related estimates (self-reported bedtime, out-of-bed time, and total bed period) than to the sleep-related estimates (self-reported try-to-sleep time, sleep onset, sleep offset, and total sleep period). For all comparisons, except the comparison between the timing of the last keystroke and self-reported bedtime, the null hypothesis that the values were similar was rejected. This suggests that, on average, there were statistically significant discrepancies between the observations from the keyboard interactions and those from the sleep diary. It is important to note that owing to the high number of observations, rather small differences could be detected using null-hypothesis testing. Therefore, these tests were complemented with equivalence tests, which indicated that the estimates obtained from the 2 modalities were, on average, equivalent when accepting an inaccuracy of 1 hour for all comparisons, except for the comparison between KAP and the self-reported total sleep period. Inspection of the descriptive statistics for the difference scores revealed that the inactive period determined by the absence of smartphone keyboard interactions was, on average, approximately 1 hour longer than the self-reported total sleep period. The findings by Druijff-van de Woestijne et al [30] also revealed a substantially longer KAP than the self-reported total sleep period (with an average of approximately 1.5 hours) among students. Interestingly, Borger et al [29] revealed a shorter rest period when estimated using smartphone touchscreen interaction monitoring than when estimated using self-reported sleep duration or actigraphy among young adults.

In this study, in contrast to the relatively large discrepancy between KAP and the self-reported total sleep period, the equivalence tests revealed that equivalence was not rejected with a margin of +30 or −30 minutes for most of the other comparisons (except for the difference between the timing of the last keystroke and self-reported try-to-sleep time and sleep onset). Earlier studies also reported average discrepancies of <30 minutes between smartphone-based estimates and self-reported or actimetry-based proxies of sleep-wake timing [29-31,56]. This suggests that the approximations of rest-activity timing rendered by smartphone interaction monitoring are very similar to those rendered by commonly used methods among free-living persons in naturalistic settings when inspecting aggregated, mean-level data. Despite the similarities on an aggregated level, this study as well as previous studies [29-31,56] also registered quite some nights that showed rather large discrepancies across the assessment modalities used, suggesting that different monitoring methods may provide rather distinct, and informative, approximations when inspecting rest-activity patterns on a day-to-day basis within persons.

Although the findings reported by Massar et al [31] showed trait-like profiles in the deviations across different assessment modalities, the ICCs in this study suggested a rather low consistency in the keyboard-derived and self-reported estimates and the deviations between these estimates across days within participants. This was particularly the case for the self-reported rest duration–related estimates, the time difference between midpoint KAP and self-reported midsleep time, and the time lag between the first keystroke and self-reported out-of-bed time, which all showed that only <20% of the variance could be explained at the person level. Day-to-day variations could not be explained by systematic differences as a function of type of day. First, the duration-related estimates did not significantly differ between weekdays and weekend days, in contrast to the timing-related estimates. Moreover, there were no systematic differences between weekdays and weekend days in the magnitude of the time differences between the keyboard-based and self-reported estimates. Interestingly, none of the associations were moderated by type of day, in contrast to the findings of the exploratory analyses reported by Druijff-van de Woestijne et al [30]. It is important to mention that this study was, in contrast to the earlier study [30], performed during the COVID-19 pandemic, and research has shown smaller distinctions in sleep timing and duration between work and work-free days because of the COVID-19–related social restrictions [57,58]. We did replicate earlier findings indicating stronger associations between the estimates obtained from smartphone interactions and those obtained from sleep diaries or actimetry on days with more regular smartphone use [29,30]. Our findings also suggested that the magnitude of the deviations for most of the sleep-related estimates as well as the time lag between the first keystroke and out-of-bed time was smaller when the keyboard was used for more hours on the previous or following day. Together, these findings suggest that the behavioral proxies obtained from smartphone interactions might be particularly powerful among persons using their smartphone (keyboard) on a regular basis during the wake episode and on days when individuals use their keyboard frequently throughout the wake episode preceding or following the rest period.

At the person level, there was quite some variation in the degree to which the keyboard-derived estimates predicted the corresponding self-reported estimates, as indicated by the random slopes. Intriguingly, the magnitude of the slope variations across students was comparable for the various associations and did not show clear metric-specific differences, except for a somewhat lower slope variation for the relationship between midpoint KAP and self-reported midsleep time. Examination of the extent to which these interindividual slope variations were dependent on chronotype, general sleep quality, and trait self-control demonstrated that general sleep quality was the most prominent moderator of the associations between the keyboard-derived and self-reported timing-related estimates. The strength of the associations between the 2 assessment modalities was lower among students who reported more disturbances in their general sleep quality. These moderations were (near) statistically significant for all associations between the keyboard-derived and self-reported timing-related markers but not for the associations between KAP and the self-reported duration-dependent markers. Research has shown that people with a lower general sleep quality are more likely to experience smartphone use addiction and show unhealthy sleep hygiene behaviors [40,41,43-45,59], including smartphone use surrounding and during sleep episodes. Although more frequent smartphone use during wake episodes could render stronger associations, as shown in this study and earlier studies [29,30], active smartphone use during sleep episodes because of fragmented sleep might underestimate the duration of the rest period and hence overestimate the timing of the rest period onset and underestimate the timing of the active period onset. Yet, inspection of the relationship between general sleep quality and KAP showed no clear relationship between the degree to which participants experienced disturbances in their general sleep quality and the duration of the prolonged keyboard inactive period at night (Multimedia Appendix 1). For most of the nights (approximately 75%), no keystrokes were registered during the self-reported sleep period, and the likelihood of keystrokes occurring during this time interval was not significantly moderated by the participants’ PSQI scores (Multimedia Appendix 1).

Chronotype did not moderate the associations between the keyboard-derived and self-reported timing-related estimates in this study. This contrasts with earlier findings, indicating stronger predictions of sleep offset by the first keystroke timing among late chronotypes than among intermediate and early chronotypes [30]. Although both samples consisted of students and had a similar chronotype range, this study was performed when COVID-19–related mandated social restrictions were in force. This might have reduced the chronotype-dependent moderations because of fewer social obligations (and the corresponding need to use an alarm) on the one hand and more mediated social interactions on the other. Intriguingly, there was a nonsignificant trend for chronotype-dependent moderation in the strength of the relationship between KAP and the self-reported total bed and total sleep periods, suggesting stronger predictions among later chronotypes. To our knowledge, this is the first time that chronotype-specific variations in the associative strength between smartphone-based estimates of the nocturnal rest duration and commonly used methods for field assessments were investigated. Replication of these findings is required, and the underlying processes and consequences need further exploration.

Although trait self-control has also been related to sleep and sleep habits, none of the associations investigated in this study were significantly moderated by trait self-control. Perhaps more interestingly, the magnitude of the deviations also showed no statistically significant associations with trait self-control, suggesting that there were no systematic variations in the timing of the last and first keystrokes surrounding KAP or the duration of KAP relative to the corresponding self-reported estimates as a function of trait self-control in the current sample. Hence, the general degree to which the students were able to inhibit their impulses and execute behaviors to achieve personal standards, adhere to social norms, and support the pursuit of their long-term goals [33,47,60] did not, on average, result in longer latencies between the last smartphone keyboard interaction and the self-reported bedtime, try-to-sleep time, and sleep onset or a longer KAP relative to the self-reported total bed period and sleep period. Chronotype also did not show statistically significant associations with the magnitude of the differences computed for the timing-related and duration-related estimates, but the magnitude of the time differences did vary as a function of general sleep quality. All comparisons showed significant or near-significant deviations, with larger discrepancies (in both directions) between the keyboard-derived and self-reported estimates. This suggests that students experiencing disturbances in sleep quality might display different behavioral strategies, ranging from avoiding active smartphone use during the period surrounding sleep to the use of the smartphone keyboard during sleep episodes. This could also explain why the likelihood of keyboard interactions occurring during sleep episodes was not significantly related to students’ general sleep quality (as assessed by the PSQI).

### Strengths, Limitations, and Implications for Future Research

In this study, we approximated the timing of the sleep and wake periods and duration of the sleep period by leveraging keyboard use behavior to estimate rest-activity patterns and compared the keyboard-derived metrics with self-reports provided the morning after the sleep episode. Although sleep diaries are a common method to monitor sleep-wake timing, subjective assessments are prone to respondent errors such as recall biases, faulty entries, and misperception. Monitoring behavior using smartphone sensors might prevent these biases [61]. Earlier studies also associated smartphone activity–based estimates (based on touchscreen interactions) with rest-activity estimates based on actimetry [29,31] and reported similar or even stronger relations. Validation of smartphone keyboard- or touchscreen-based metrics for rest-activity monitoring against polysomnography (preferably obtained in the field), the gold-standard technique to study sleep [28], would be an important next step. Although the use of the CSD enabled us to distinguish between different bed-related and sleep-related metrics of the onset of the rest and active periods, polysomnography could more accurately classify events as sleep or wake and would allow the inspection of the occurrence of smartphone interactions during different sleep stages. In fact, there were quite some nights on which keyboard interactions occurred after self-reported sleep onset and before self-reported sleep offset (approximately 25% of the nights), which questions the accuracy of these self-reported estimates. The likelihood of touchscreen interactions occurring after sleep onset and before sleep offset might be even higher [29].

The monitoring of keyboard use behavior enables the unobtrusive monitoring of cognitively engaging events surrounding sleep but does not monitor more passive smartphone use (such as watching movies). This might have resulted in an underestimation of the timing of the rest period onset and an overestimation of the active period onset, as reflected in the average deviations. As indicated earlier, KAP was larger than the self-reported total sleep period for the vast majority of the nights. This finding contrasts with the underestimation of sleep duration based on touchscreen interaction monitoring versus self-reports [29]. The extent to which these different findings can be attributed to the method used to monitor smartphone interactions (keyboard interactions vs touchscreen interactions) as well as the algorithm to determine the onset of the rest and active periods remains to be tested. Similar to the method used by Druijff-van de Woestijne et al [30], we used a simple algorithm to estimate rest based on prolonged keystroke inactivity. Future research could consider more advanced modeling of keyboard interactions to further improve the predictions or integrating multisensor data to include additional behavioral markers and features of the ambient environment to facilitate rest period detection [62,63]. Nonetheless, the current and earlier findings [30] showed that a single event, such as the last keystroke before a prolonged period of keyboard inactivity, can explain a rather large part of the variance in the self-reported onset timing of the rest and active periods.

The current research outcomes replicated the association between keyboard interactions and self-reported rest and activity onset timing [30] in a relatively large sample. An innovation of this study is that we also inspected interindividual differences in the associations and deviations between these 2 assessment modalities as a function of person-level variables related to sleep and sleep habits. Similar to a previous study [30], the current sample consisted of first-year students. This is an important target population to consider, as young adults are particularly susceptible to sleep disturbances, including circadian rhythm disorder [20]. However, the recruitment of young adults in higher education with a restricted age range may also question the generalizability and sustainability of the prediction when applying the same method among the general population or specific groups such as clinical populations. The extent to which the results can be translated to a more heterogeneous sample with stronger variability in demographics and contextual factors remains to be investigated in future research.

There are considerable differences between adolescents and older adults. Whereas the younger generations are digital natives, older adults have only learned to use smartphones in adulthood. However, from the few statistics that do report on phone use among older populations, it appears that although smartphone use decreases with age, this negative trend is rather subtle [64-66]. Hence, the usability of these metrics may also be substantial for older adults. However, it is important to note that although the decrease in smartphone use might be small, app use and dynamics in smartphone use likely differ to a larger extent as a function of age [64-66]. A large-scale study using sleep-tracking app data also revealed shorter sleep duration and lower sleep quality among younger individuals than among older individuals [67]. Moreover, earlier findings revealed distinct sleep and perisleep smartphone use patterns among a subgroup of participants that was characterized by young adults, mainly students [31], compared with, for example, somewhat older participants. The investigation of interindividual variability in the current student sample suggested that the estimation of rest-activity cycles by means of keyboard interaction monitoring might render slightly less accurate results for students with disturbances in sleep quality, at least when compared with self-reported estimates. The replication of the current results among a larger sample with a more diverse age range and including clinical patients with sleep disorders is needed to increase the generalizability of the findings.

It should be noted that, to our knowledge, this is the first time that the associations and deviations between estimates of rest-activity patterns obtained from smartphone interaction monitoring and those obtained from sleep diaries are contrasted between individuals as a function of their general sleep quality and trait self-control. More research is needed to further elucidate the role of general sleep quality in the associations between smartphone interaction monitoring and commonly used methods. However, the current findings do suggest that person-tuned instead of population-based models for predicting rest-activity patterns based on smartphone interactions are likely more accurate. It should also be acknowledged that the method adopted is suitable only for daytime active persons and cannot detect daytime naps. The translation of the findings to night- or rotating-shift workers and older or clinical populations requires further consideration.

Another important consideration is the fact that the use of screen technology (including smartphones) in the late subjective evening has been linked to disturbances in sleep [68-71], suggesting that we may not want to strive to minimize deviations as smartphone use may hinder a good night of sleep. Most studies investigating the association between smartphone use and sleep used self-reports to monitor smartphone use and, hence, are vulnerable to respondent errors [72,73]. Moreover, most studies assessed associations between smartphone use and sleep-related markers cross-sectionally, hindering insights into variances within participants over time and limiting the establishment of causal relationships [72]. Although used as a monitoring tool to approximate rest-activity patterns in this study, smartphone interaction monitoring would also allow for fine-grained assessment of the use of digital media surrounding sleep. In fact, insights into the interference of smartphone use with sleep and rest-activity patterns might inform the development of person-tuned strategies to promote a healthy sleep hygiene. Moreover, low-cost tools for fostering long-term rest-activity pattern monitoring could serve as a preventive strategy and an early-warning system in the domains of sleep and health. Yet, generalization of the current and previous findings as well as optimization of the accuracy of the estimates across a wide variety of potential users would benefit large-scale adoption.

### Conclusions

In summary, we replicated earlier findings on the potential of smartphone interaction monitoring to serve as a method for deriving behavioral proxies to approximate rest-activity patterns among students. This study complemented these earlier findings by suggesting that the accuracy of the behavioral proxies might be lower among students with disturbances in their general sleep quality. The generalization and underlying process of these findings require further investigation.

## Appendix

Multimedia Appendix 1 An overview of the response rates per day and the descriptive statistics and visual inspection of the time differences between the keyboard-derived and the corresponding self-reported estimates. Moreover, the results of the multilevel models testing moderations by hours with smartphone use and type of day as well as chronotype, general sleep quality, and trait self-control are presented.

## Abbreviations

µMCTQ: Ultrashort Munich Chronotype Questionnaire
CSD: Consensus Sleep Diary
ICC: intraclass correlation
KAP: keystroke-absence period
MLM: multilevel model
PSQI: Pittsburg Sleep Quality Index
TSC: Trait Self-Control Scale

## References

[1] Alhola P, Polo-Kantola P (2007). Sleep deprivation: impact on cognitive performance. Neuropsychiatr Dis Treat.

[2] Alvaro PK, Roberts RM, Harris JK (2013). A systematic review assessing bidirectionality between sleep disturbances, anxiety, and depression. Sleep.

[3] Chaput J, Dutil C, Featherstone R, Ross R, Giangregorio L, Saunders TJ, Janssen I, Poitras VJ, Kho ME, Ross-White A, Zankar S, Carrier J (2020). Sleep timing, sleep consistency, and health in adults: a systematic review. Appl Physiol Nutr Metab.

[4] Lo JC, Groeger JA, Cheng GH, Dijk D, Chee MW (2016). Self-reported sleep duration and cognitive performance in older adults: a systematic review and meta-analysis. Sleep Med.

[5] Lovato N, Gradisar M (2014). A meta-analysis and model of the relationship between sleep and depression in adolescents: recommendations for future research and clinical practice. Sleep Med Rev.

[6] Walker MP (2008). Cognitive consequences of sleep and sleep loss. Sleep Med.

[7] Wardle-Pinkston S, Slavish DC, Taylor DJ (2019). Insomnia and cognitive performance: a systematic review and meta-analysis. Sleep Med Rev.

[8] Durmer JS, Dinges DF (2005). Neurocognitive consequences of sleep deprivation. Semin Neurol.

[9] Genzel L, Ahrberg K, Roselli C, Niedermaier S, Steiger A, Dresler M, Roenneberg T (2013). Sleep timing is more important than sleep length or quality for medical school performance. Chronobiol Int.

[10] Killgore W (2010). Effects of sleep deprivation on cognition. Prog Brain Res.

[11] Wittmann M, Dinich J, Merrow M, Roenneberg T (2006). Social jetlag: misalignment of biological and social time. Chronobiol Int.

[12] Pilz LK, Keller LK, Lenssen D, Roenneberg T (2018). Time to rethink sleep quality: PSQI scores reflect sleep quality on workdays. Sleep.

[13] Vitale JA, Roveda E, Montaruli A, Galasso L, Weydahl A, Caumo A, Carandente F (2015). Chronotype influences activity circadian rhythm and sleep: differences in sleep quality between weekdays and weekend. Chronobiol Int.

[14] Akerstedt T, Axelsson J, Lekander M, Orsini N, Kecklund G (2014). Do sleep, stress, and illness explain daily variations in fatigue? A prospective study. J Psychosom Res.

[15] Narmandakh A, Oldehinkel AJ, Masselink M, de Jonge P, Roest AM (2021). Affect, worry, and sleep: between- and within-subject associations in a diary study. J Affective Disorder Reports.

[16] Lemola S, Ledermann T, Friedman EM (2013). Variability of sleep duration is related to subjective sleep quality and subjective well-being: an actigraphy study. PLoS One.

[17] Lancel M, van Veen MM, Verbeek IH (2020). [Sleep: the basis of a healthy lifestyle]. Tijdschr Psychiatr.

[18] Guarana CL, Ryu JW, O'Boyle EH, Lee J, Barnes CM (2021). Sleep and self-control: a systematic review and meta-analysis. Sleep Med Rev.

[19] Hisler GC, Krizan Z, DeHart T (2019). Does stress explain the effect of sleep on self-control difficulties? A month-long daily diary study. Pers Soc Psychol Bull.

[20] Holstein BE, Ammitzbøll J, Damsgaard MT, Pant SW, Pedersen TP, Skovgaard AM (2020). Difficulties falling asleep among adolescents: social inequality and time trends 1991-2018. J Sleep Res.

[21] Ghekiere A, Van Cauwenberg J, Vandendriessche A, Inchley J, Gaspar de Matos M, Borraccino A, Gobina I, Tynjälä J, Deforche B, De Clercq B (2019). Trends in sleeping difficulties among European adolescents: are these associated with physical inactivity and excessive screen time?. Int J Public Health.

[22] Grandner M (2019). Epidemiology of insufficient sleep and poor sleep quality. Sleep and Health.

[23] Jahrami H, BaHammam AS, Bragazzi NL, Saif Z, Faris M, Vitiello MV (2021). Sleep problems during the COVID-19 pandemic by population: a systematic review and meta-analysis. J Clin Sleep Med.

[24] Mattingly SM, Grover T, Martinez GJ, Aledavood T, Robles-Granda P, Nies K, Striegel A, Mark G (2021). The effects of seasons and weather on sleep patterns measured through longitudinal multimodal sensing. NPJ Digit Med.

[25] Leypunskiy E, Kıcıman E, Shah M, Walch OJ, Rzhetsky A, Dinner AR, Rust MJ (2018). Geographically resolved rhythms in twitter use reveal social pressures on daily activity patterns. Curr Biol.

[26] Roenneberg T (2017). Twitter as a means to study temporal behaviour. Curr Biol.

[27] Loock A, Khan Sullivan A, Reis C, Paiva T, Ghotbi N, Pilz LK, Biller AM, Molenda C, Vuori-Brodowski MT, Roenneberg T, Winnebeck EC (2021). Validation of the Munich actimetry sleep detection algorithm for estimating sleep-wake patterns from activity recordings. J Sleep Res.

[28] Perez-Pozuelo I, Zhai B, Palotti J, Mall R, Aupetit M, Garcia-Gomez JM, Taheri S, Guan Y, Fernandez-Luque L (2020). The future of sleep health: a data-driven revolution in sleep science and medicine. NPJ Digit Med.

[29] Borger JN, Huber R, Ghosh A (2019). Capturing sleep-wake cycles by using day-to-day smartphone touchscreen interactions. NPJ Digit Med.

[30] Druijff-van DW, McConchie H, de Kort Y, Licitra G, Zhang C, Overeem S, Smolders K (2021). Behavioural Biometrics: using smartphone keyboard activity as a proxy for rest-activity patterns. J Sleep Res.

[31] Massar SA, Chua XY, Soon CS, Ng AS, Ong JL, Chee NI, Lee TS, Ghosh A, Chee MW (2021). Trait-like nocturnal sleep behavior identified by combining wearable, phone-use, and self-report data. NPJ Digit Med.

[32] Levenson JC, Shensa A, Sidani JE, Colditz JB, Primack BA (2017). Social media use before bed and sleep disturbance among young adults in the United States: a nationally representative study. Sleep.

[33] Exelmans L (2019). Electronic media use and sleep: a self-control perspective. Curr Sleep Medicine Rep.

[34] Pilcher JJ, Morris DM, Donnelly J, Feigl HB (2015). Interactions between sleep habits and self-control. Front Hum Neurosci.

[35] Hisler G, Kri?an Z (2019). Dynamics between sleep and self-control. Sleep, Personality, and Social Behavior.

[36] Przepiórka A, Błachnio A, Siu NY (2019). The relationships between self-efficacy, self-control, chronotype, procrastination and sleep problems in young adults. Chronobiol Int.

[37] Brick CA, Seely DL, Palermo TM (2010). Association between sleep hygiene and sleep quality in medical students. Behav Sleep Med.

[38] Kroese FM, Evers C, Adriaanse MA, de Ridder DT (2016). Bedtime procrastination: a self-regulation perspective on sleep insufficiency in the general population. J Health Psychol.

[39] Nauts S, Kroese FM (2017). The role of self-control in sleep behavior. Routledge International Handbook of Self-Control in Health and Well-Being.

[40] Woods HC, Scott H (2016). #Sleepyteens: social media use in adolescence is associated with poor sleep quality, anxiety, depression and low self-esteem. J Adolesc.

[41] Barber L, Grawitch MJ, Munz DC (2013). Are better sleepers more engaged workers? A self-regulatory approach to sleep hygiene and work engagement. Stress Health.

[42] Todd J, Mullan B (2013). The role of self-regulation in predicting sleep hygiene in university students. Psychol Health Med.

[43] Christensen MA, Bettencourt L, Kaye L, Moturu ST, Nguyen KT, Olgin JE, Pletcher MJ, Marcus GM (2016). Direct measurements of smartphone screen-time: relationships with demographics and sleep. PLoS One.

[44] Exelmans L, Van den Bulck J (2016). Bedtime mobile phone use and sleep in adults. Soc Sci Med.

[45] Tavernier R, Willoughby T (2014). Sleep problems: predictor or outcome of media use among emerging adults at university?. J Sleep Res.

[46] Carney CE, Buysse DJ, Ancoli-Israel S, Edinger JD, Krystal AD, Lichstein KL, Morin CM (2012). The consensus sleep diary: standardizing prospective sleep self-monitoring. Sleep.

[47] Tangney JP, Baumeister RF, Boone AL (2004). High self-control predicts good adjustment, less pathology, better grades, and interpersonal success. J Pers.

[48] Buysse DJ, Reynolds CF, Monk TH, Berman SR, Kupfer DJ (1989). The Pittsburgh sleep quality index: a new instrument for psychiatric practice and research. Psychiatry Res.

[49] Ghotbi N, Pilz LK, Winnebeck EC, Vetter C, Zerbini G, Lenssen D, Frighetto G, Salamanca M, Costa R, Montagnese S, Roenneberg T (2020). The µMCTQ: an ultra-short version of the Munich ChronoType questionnaire. J Biol Rhythms.

[50] Krupp LB, LaRocca N G, Muir-Nash J, Steinberg A D (1989). The fatigue severity scale. Application to patients with multiple sclerosis and systemic lupus erythematosus. Arch Neurol.

[51] Rietberg MB, Van Wegen EE, Kwakkel G (2010). Measuring fatigue in patients with multiple sclerosis: reproducibility, responsiveness and concurrent validity of three Dutch self-report questionnaires. Disabil Rehabil.

[52] Akerstedt T, Gillberg M (1990). Subjective and objective sleepiness in the active individual. Int J Neurosci.

[53] Zhang C, Smolders KCHJ, Lakens D, IJsselsteijn WA (2018). Two experience sampling studies examining the variation of self-control capacity and its relationship with core affect in daily life. Journal of Research in Personality.

[54] Ciarocco N, Twenge J, Muraven M, Tice D (2004). Measuring state self-control: reliability, validity, and correlations with physical and psychological stress. San Diego State University.

[55] Lakens D, Scheel A, Isager P (2018). Equivalence testing for psychological research: a tutorial. Advance Methods Practice Psychological Sci.

[56] Huber R, Ghosh A (2021). Large cognitive fluctuations surrounding sleep in daily living. iScience.

[57] Blume C, Schmidt MH, Cajochen C (2020). Effects of the COVID-19 lockdown on human sleep and rest-activity rhythms. Curr Biol.

[58] Korman M, Tkachev V, Reis C, Komada Y, Kitamura S, Gubin D, Kumar V, Roenneberg T (2020). COVID-19-mandated social restrictions unveil the impact of social time pressure on sleep and body clock. Sci Rep.

[59] Qanash S, Al-Husayni F, Falata H, Halawani O, Jahra E, Murshed B, Alhejaili F, Ghabashi A, Alhashmi H (2021). Effect of electronic device addiction on sleep quality and academic performance among health care students: cross-sectional study. JMIR Med Educ.

[60] Baumeister RF, Vohs KD, Tice DM (2016). The strength model of self-control. Curr Dir Psychol Sci.

[61] Fischer F, Kleen S (2021). Possibilities, problems, and perspectives of data collection by mobile apps in longitudinal epidemiological studies: scoping review. J Med Internet Res.

[62] Saeb S, Cybulski TR, Schueller SM, Kording KP, Mohr DC (2017). Scalable passive sleep monitoring using mobile phones: opportunities and obstacles. J Med Internet Res.

[63] Martinez G, Mattingly S, Young J, Faust L, Dey AK, Campbell AT, De Choudhury M, Mirjafari S, Nepal SK, Robles-Granda P, Saha K, Striegel AD (2020). Improved sleep detection through the fusion of phone agent and wearable data streams. Proceedings of the IEEE International Conference on Pervasive Computing and Communications Workshops (PerCom Workshops).

[64] Andone I, Blaszkiewicz K, Eibes M, Trendafilov B, Markowetz A, Montag C (2016). How age and gender affect smartphone usage. Proceedings of the 2016 ACM International Joint Conference on Pervasive and Ubiquitous Computing: Adjunct.

[65] Montag C, Błaszkiewicz K, Sariyska R, Lachmann B, Andone I, Trendafilov B, Eibes M, Markowetz A (2015). Smartphone usage in the 21st century: who is active on WhatsApp?. BMC Res Notes.

[66] Ceolini E, Kock R, Band GP, Stoet G, Ghosh A (2022). Temporal clusters of age-related behavioral alterations captured in smartphone touchscreen interactions. iScience.

[67] Robbins R, Affouf M, Seixas A, Beaugris L, Avirappattu G, Jean-Louis G (2020). Four-year trends in sleep duration and quality: a longitudinal study using data from a commercially available sleep tracker. J Med Internet Res.

[68] Cain N, Gradisar M (2010). Electronic media use and sleep in school-aged children and adolescents: a review. Sleep Med.

[69] Hale L, Guan S (2015). Screen time and sleep among school-aged children and adolescents: a systematic literature review. Sleep Med Rev.

[70] Levenson JC, Shensa A, Sidani JE, Colditz JB, Primack BA (2016). The association between social media use and sleep disturbance among young adults. Prev Med.

[71] Varghese NE, Santoro E, Lugo A, Madrid-Valero JJ, Ghislandi S, Torbica A, Gallus S (2021). The role of technology and social media use in sleep-onset difficulties among Italian adolescents: cross-sectional study. J Med Internet Res.

[72] Yang J, Fu X, Liao X, Li Y (2020). Association of problematic smartphone use with poor sleep quality, depression, and anxiety: a systematic review and meta-analysis. Psychiatry Res.

[73] Mac Cárthaigh S, Griffin C, Perry J (2020). The relationship between sleep and problematic smartphone use among adolescents: a systematic review. Developmental Rev.

