# Supplementary material for: Smartphone Keyboard Interaction Monitoring as an Unobtrusive Method to Approximate Rest-Activity Patterns: Experience Sampling Study Investigating Interindividual and Metric-Specific Variations
Source: J Med Internet Res. 2023 Apr 7;25:e38066. doi: 10.2196/38066 (PMC10131989; doi:10.2196/38066)

# Supplemental Materials -Smartphone keyboard interaction monitoring as an unobtrusive method to approximate rest-activity patterns: Inter-individual and metric-specific variations

## Response Rates per Participant and per Assessment Day

Figure S.1 displays histograms of the response rates and the total number of completed diaries per day.

**Figure S.1.** Histograms of the response rates and the number of completed diaries per day.


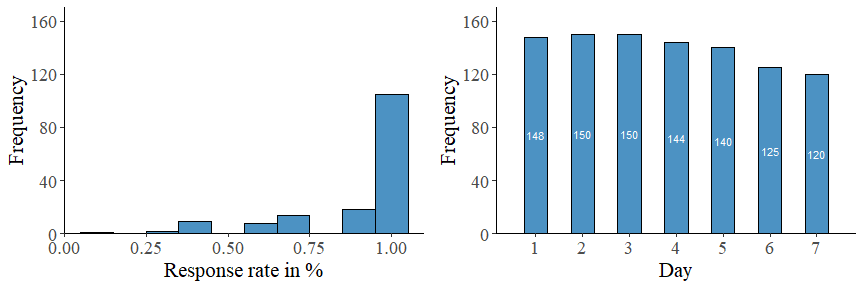


## Descriptive Statistics of the Time Differences between the Keyboard-derived and the Corresponding Self-reported Estimates

Table S.1. shows the descriptive statistics for the difference scores between the keyboard-derived and the corresponding self-reported estimates. The ICCs reported in this table are based on the unconditional MLMs with the time differences between the two assessment modalities as outcome parameters (separate models per difference score). The ICCs of these deltas indicate that most of the variance in the differences between the keyboard-derived and self-reported estimates could be explained at the first level (i.e., Day level), with 15-34% of the variance explained at the second level (i.e., Participant level).

**Table S.1.** Descriptive statistics of difference scores between keyboard-derived and self-reported estimates of rest-activity timing and duration (in hrs)

|  | Mean | SD | Min | Max | Skewness | Kurtosis | ICC |
| --- | --- | --- | --- | --- | --- | --- | --- |
| Last keystroke - bedtime | 0.20 | 1.13 | -6.36 | 6.12 | -0.15 | 8.71 | .26 |
| Last keystroke - try-to-sleep time | -0.35 | 1.11 | -6.36 | 5.32 | -0.96 | 9.74 | .27 |
| Last keystroke - sleep onset | -0.62 | 1.11 | -6.44 | 5.32 | -0.85 | 9.63 | .30 |
| Midpoint KAP - mid-sleep time | -0.13 | 0.73 | -4.16 | 3.31 | -0.13 | 7.35 | .15 |
| First keystroke - sleep offset | 0.38 | 1.19 | -4.54 | 8.11 | 0.91 | 11.02 | .24 |
| First keystroke – out-of-bed time | -0.13 | 1.20 | -5.24 | 7.86 | 0.67 | 11.34 | .19 |
| KAP - total sleep period | -0.34 | 1.74 | -6.64 | 7.55 | 0.41 | 5.80 | .29 |
| KAP - total bed period | 0.99 | 1.76 | -5.50 | 10.48 | 0.80 | 6.34 | .34 |

*Note.* ICC stands for intra-class correlation, which was determined with the corresponding unconditional (intercepts-only) model, and represents the variance explained at the Participant level.

## Visual Inspection of Agreement and Differences between Keyboard-derived and Self-reported Estimates as a Function of their Average Value

**Figure S.2.** Bland-Altman plots of rest-activity pattern estimates derived from the sleep diary and keystroke logging data.


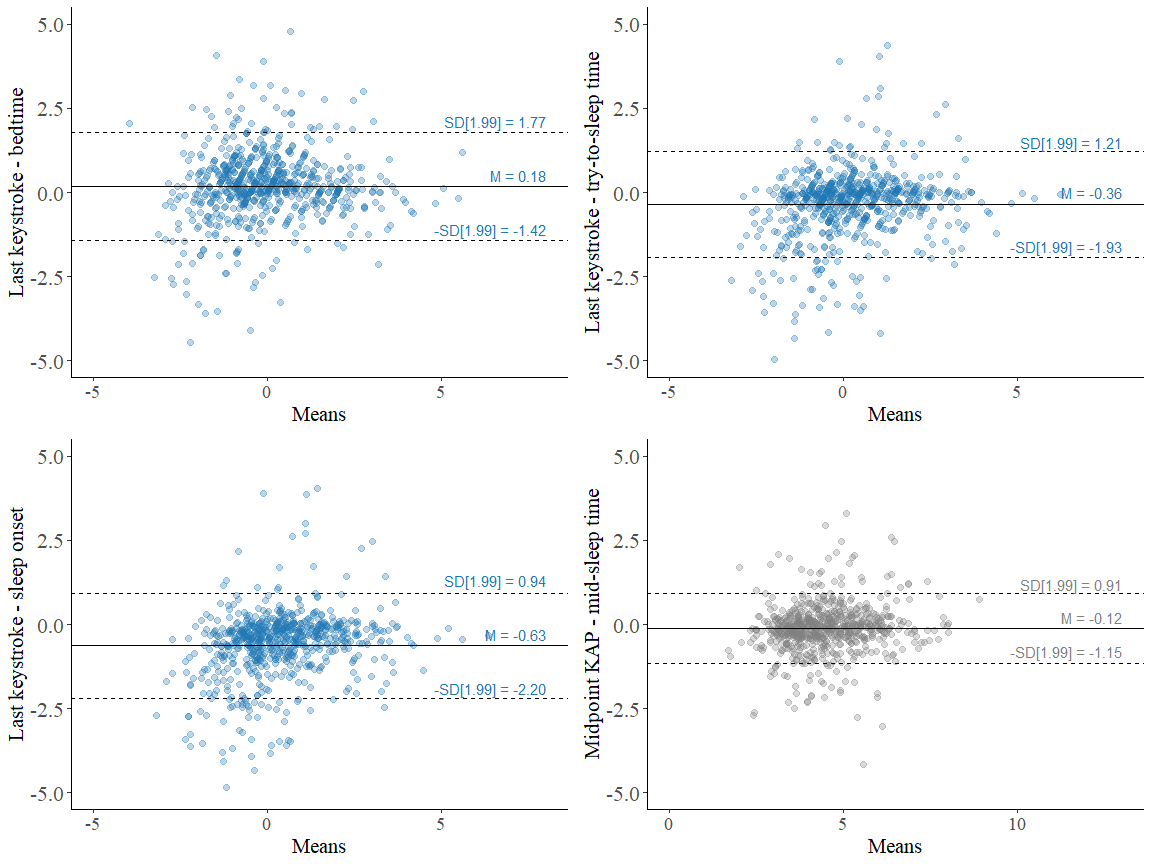


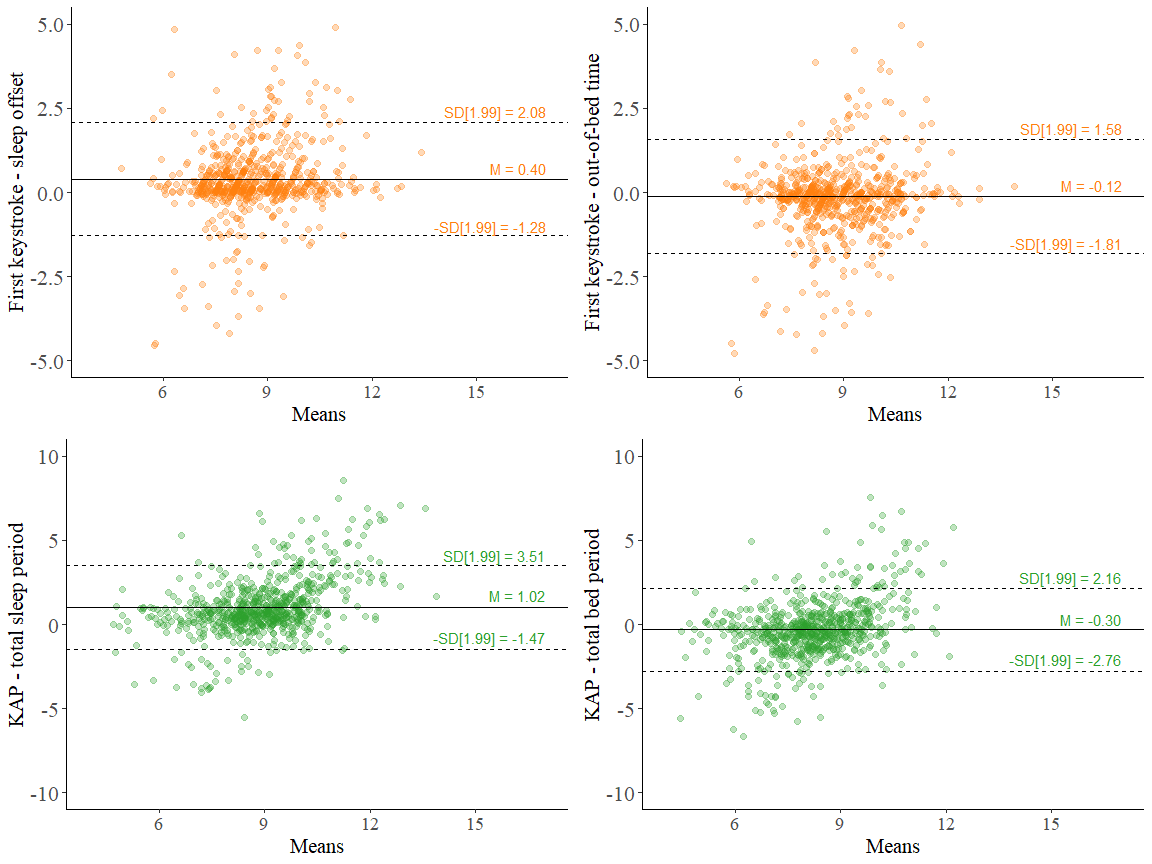


The means on the x-axis display the mean value of the putative time or duration as assessed with the keyboard-derived and self-reported metrics. Values smaller than zero represent a later timing or longer duration for the self-reported vs. keyboard-derived estimates, and positive values reflect an earlier timing or shorter duration for the self-reported vs. keyboard-derived estimates. Differences are displayed in hours.

## Moderations in Relational Strength between Keyboard-derived and Self-reported Estimates by Hours with Smartphone Use and Type of Day

Tables S.2-S.9 present the results of the MLMs with the first-level predictors included as potential moderators of the association between the keyboard-derived and self-reported estimates.

**Table S.2.** Results of multilevel model for self-reported bedtime with last keystroke, previous hours with keyboard activity, and type of day as predictors.

| Predictor | Beta | .5% | 99.5% | t | df | p |
| --- | --- | --- | --- | --- | --- | --- |
| Last keystroke - participant mean | 0.30 | 0.20 | 0.40 | 7.83 | 129.9 | <.001 |
| Last keystroke | 0.58 | 0.51 | 0.65 | 20.79 | 609.1 | <.001 |
| Previous hours with keyboard activity | <.01 | -0.06 | 0.06 | -0.16 | 606.7 | .875 |
| Weekend | 0.20 | 0.06 | 0.33 | 3.58 | 616.4 | <.001 |
| Last keystroke * Previous hours with keyboard activity | 0.09 | 0.03 | 0.15 | 3.97 | 651.1 | <.001 |
| Last keystroke * Weekend | 0.06 | -0.08 | 0.20 | 1.14 | 652.7 | .257 |

**Table S.3.** Results of multilevel model for self-reported try-to-sleep time with last keystroke, previous hours with keyboard activity, and type of day as predictors.

| Predictor | Beta | .5% | 99.5% | t | df | p |
| --- | --- | --- | --- | --- | --- | --- |
| Last keystroke - participant mean | 0.30 | 0.20 | 0.40 | 7.69 | 130.0 | <.001 |
| Last keystroke | 0.61 | 0.53 | 0.68 | 21.68 | 609.4 | <.001 |
| Previous hours with keyboard activity | -0.01 | -0.07 | 0.04 | -0.66 | 606.9 | .513 |
| Weekend | 0.20 | 0.06 | 0.34 | 3.71 | 616.7 | <.001 |
| Last keystroke * Previous hours with keyboard activity | 0.08 | 0.02 | 0.14 | 3.53 | 651.7 | <.001 |
| Last keystroke * Weekend | -0.02 | -0.16 | 0.12 | -0.43 | 653.3 | .669 |

**Table S.4.** Results of multilevel model for self-reported sleep onset with last keystroke, previous hours with keyboard activity, and type of day as predictors.

| Predictor | Beta | .5% | 99.5% | t | Df | p |
| --- | --- | --- | --- | --- | --- | --- |
| Last keystroke - participant mean | 0.28 | 0.18 | 0.38 | 7.06 | 129.6 | <.001 |
| Last keystroke | 0.62 | 0.55 | 0.69 | 22.49 | 605.8 | <.001 |
| Previous hours with keyboard activity | -0.04 | -0.10 | 0.02 | -1.76 | 604.7 | .079 |
| Weekend | 0.15 | 0.01 | 0.29 | 2.76 | 613.3 | .006 |
| Last keystroke * Previous hours with keyboard activity | 0.08 | 0.02 | 0.14 | 3.38 | 645.9 | .001 |
| Last keystroke * Weekend | -0.04 | -0.18 | 0.09 | -0.81 | 647.2 | .416 |

**Table S.5.** Results of multilevel model for self-reported sleep offset with first keystroke, hours with keyboard activity, and type of day as predictors.

| Predictor | Beta | .5% | 99.5% | t | df | p |
| --- | --- | --- | --- | --- | --- | --- |
| First keystroke - participant mean | 0.35 | 0.23 | 0.46 | 7.84 | 127.0 | <.001 |
| First keystroke | 0.43 | 0.35 | 0.51 | 12.62 | 626.5 | <.001 |
| Hours with keyboard activity | <.01 | -0.07 | 0.07 | -0.02 | 614.3 | .985 |
| Weekend | 0.31 | 0.15 | 0.47 | 4.96 | 615.9 | <.001 |
| First keystroke * Hours with keyboard activity | 0.12 | 0.06 | 0.19 | 5.22 | 657.8 | <.001 |
| First keystroke * Weekend | 0.06 | -0.10 | 0.21 | 0.92 | 670.4 | .356 |

**Table S.6.** Results of multilevel model for self-reported out-of-bed time with first keystroke, hours with keyboard activity, and type of day as predictors.

| Predictor | Beta | .5% | 99.5% | t | df | p |
| --- | --- | --- | --- | --- | --- | --- |
| First keystroke - participant mean | 0.32 | 0.21 | 0.42 | 7.75 | 124.3 | <.001 |
| First keystroke | 0.47 | 0.38 | 0.56 | 13.01 | 639.8 | <.001 |
| Hours with keyboard activity | <.01 | -0.07 | 0.07 | 0.03 | 622.5 | .977 |
| Weekend | 0.25 | 0.08 | 0.42 | 3.74 | 626.1 | <.001 |
| First keystroke * Hours with keyboard activity | 0.13 | 0.07 | 0.20 | 5.16 | 677.3 | <.001 |
| First keystroke * Weekend | 0.04 | -0.13 | 0.21 | 0.61 | 689.7 | .539 |

**Table S.7.** Results of multilevel model for self-reported mid-sleep timing with midpoint KAP, previous hours with keyboard activity, hours with keyboard activity, and type of day as predictors.

| Predictor | Beta | .5% | 99.5% | t | df | p |
| --- | --- | --- | --- | --- | --- | --- |
| Midpoint KAP - participant mean | 0.43 | 0.35 | 0.51 | 13.87 | 124.3 | <.001 |
| Midpoint KAP | 0.55 | 0.48 | 0.62 | 20.29 | 621.7 | <.001 |
| Previous hours with keyboard activity | -0.02 | -0.07 | 0.03 | -0.88 | 607.3 | .378 |
| Hours with keyboard activity | <.01 | -0.05 | 0.05 | -0.10 | 619.4 | .921 |
| Weekend | 0.18 | 0.05 | 0.31 | 3.52 | 618.7 | <.001 |
| Midpoint KAP * Previous hours with keyboard activity | -0.01 | -0.06 | 0.04 | -0.58 | 665.5 | .560 |
| Midpoint KAP * Hours with keyboard activity | 0.10 | 0.05 | 0.15 | 5.29 | 660.1 | <.001 |
| Midpoint KAP * Weekend | 0.09 | -0.05 | 0.22 | 1.71 | 667.4 | .087 |

**Table S.8**. Results of multilevel model for self-reported total sleep period with KAP, previous hours with keyboard activity, hours with keyboard activity, and type of day as predictors.

| Predictor | Beta | .5% | 99.5% | t | df | p |
| --- | --- | --- | --- | --- | --- | --- |
| KAP - participant mean | 0.05 | -0.06 | 0.16 | 1.16 | 123.3 | .250 |
| KAP | 0.47 | 0.36 | 0.57 | 10.82 | 625.2 | <.001 |
| Previous hours with keyboard activity | 0.03 | -0.05 | 0.11 | 1.06 | 608.2 | .290 |
| Hours with keyboard activity | -0.01 | -0.10 | 0.07 | -0.36 | 621.2 | .720 |
| Weekend | 0.12 | -0.07 | 0.32 | 1.63 | 619.9 | .104 |
| KAP * Previous hours with keyboard activity | 0.06 | -0.01 | 0.14 | 2.19 | 679.7 | .029 |
| KAP * Hours with keyboard activity | 0.18 | 0.10 | 0.26 | 5.573 | 682.2 | <.001 |
| KAP * Weekend | 0.05 | -0.13 | 0.23 | 0.69 | 668.8 | .491 |

**Table S.9.** Results of multilevel model for self-reported total bed period with KAP, previous hours with keyboard activity, hours with keyboard activity, and type of day as predictors.

| Predictor | Beta | .5% | 99.5% | t | Df | p |
| --- | --- | --- | --- | --- | --- | --- |
| KAP - participant mean | 0.11 | <.01 | 0.23 | 2.47 | 127.9 | .015 |
| KAP | 0.44 | 0.33 | 0.54 | 10.31 | 628.4 | <.001 |
| Previous hours with keyboard activity | -0.02 | -0.10 | 0.06 | -0.52 | 611.1 | .607 |
| Hours with keyboard activity | <.01 | -0.09 | 0.09 | -0.05 | 623.7 | .964 |
| Weekend | <.01 | -0.20 | 0.19 | -0.06 | 623.3 | .954 |
| KAP * Previous hours with keyboard activity | 0.02 | -0.05 | 0.10 | 0.84 | 682.8 | .402 |
| KAP * Hours with keyboard activity | 0.16 | 0.08 | 0.24 | 5.07 | 681.5 | <.001 |
| KAP * Weekend | 0.13 | -0.06 | 0.31 | 1.75 | 671.7 | .081 |

## Keyboard-derived and Self-reported Estimates as Function of Type of Day

Figure S.3 shows the Boxplots of the keyboard-derived and self-reported estimates for weekdays and weekend days, and Table S.10 presents the results of MLMs investigating differences in the keyboard-derived and self-reported estimates between weekdays and weekend days. In these MLMs, only type of day was included as fixed factor and the various timing and duration-related estimates were included as outcome parameters (separate model per variable).

**Figure S.3.** Boxplots of rest-activity pattern estimates as function of type of day.


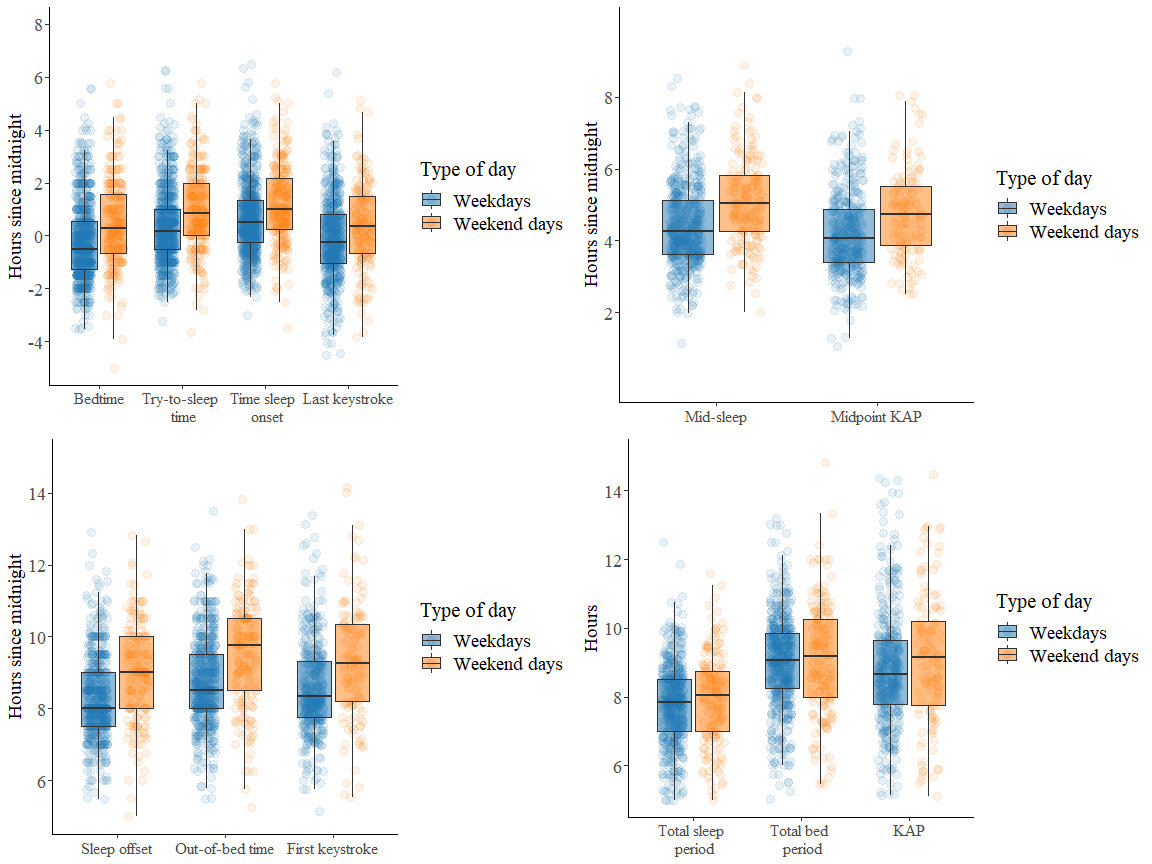


**Table S.10.** Results of multilevel models for keyboard-derived and self-reported estimates regressed on type of day.

| Outcome parameter | Beta | .5% | 99.5% | t | df | p |
| --- | --- | --- | --- | --- | --- | --- |
| Bedtime | 0.45 | 0.27 | 0.62 | 6.53 | 726.5 | <.001 |
| Try-to-sleep time | 0.44 | 0.27 | 0.62 | 6.47 | 725.7 | <.001 |
| Sleep onset | 0.41 | 0.23 | 0.59 | 5.97 | 725.6 | <.001 |
| Last keystroke | 0.31 | 0.11 | 0.51 | 4.06 | 626.2 | <.001 |
| Mid-sleep time | 0.56 | 0.40 | 0.73 | 8.74 | 711.3 | <.001 |
| Midpoint KAP | 0.48 | 0.29 | 0.67 | 6.53 | 624.6 | <.001 |
| Sleep offset | 0.58 | 0.42 | 0.75 | 9.01 | 718.9 | <.001 |
| Out-of-bed time | 0.54 | 0.37 | 0.72 | 7.92 | 723.1 | <.001 |
| First keystroke | 0.50 | 0.31 | 0.68 | 6.79 | 621.6 | <.001 |
| Total bed period | 0.03 | -0.16 | 0.22 | 0.42 | 725.0 | .672 |
| Total sleep period | 0.11 | -0.08 | 0.31 | 1.47 | 721.2 | .143 |
| KAP | 0.11 | -0.09 | 0.31 | 1.45 | 624.8 | .149 |

*Note.* A positive parameter estimates indicates a later timing or longer duration on weekend days vs. weekdays.

## Moderations in the Time Differences between Keyboard-derived and Self-reported Estimates by Hours with Smartphone Use and Type of Day

MLMs with the absolute time differences between the keyboard-derived and the corresponding self-reported estimates as outcome parameters and the number of hours with keyboard activity and type of day were performed to test whether the magnitude of the differences was moderated by these first-level predictors. Results of these models revealed that type of day did not significantly moderate the extent to which the two assessment modalities deviated (all p > .05). The time differences between the last keystroke and the self-reported bedtime and try-to-sleep time were also not significantly associated with the relative number of hours with keyboard activity before the rest period (p = .34 and p = .06). In contrast, there was a non-significant trend for smaller time differences between the timing of the last keystroke and self-reported sleep onset when participants used their keyboard during more hours on days before the rest period than their week average (Beta = -0.08 [-0.16,0.01], t(603) = -2.24, p = .03). The timing of the first keystroke was closer to the self-reported sleep offset when participants had more hours with keystroke activity during the following day (Beta = -0.15 [-0.25,-0.06], t(617) = -4.16, p = <.001). A similar, but non-significant trend was found for self-reported out-of-bed time (Beta = -0.1 [-0.19,<.01], t(625) = -2.54, p = 0.011). The time difference between midpoint KAP and self-reported mid-sleep time as well as the time difference between KAP and self-reported total sleep period was dependent on the relative number of hours with keystroke activity on the following day (Beta = -0.14 [-0.24,-0.04], t(631) = -3.69, p = <.001 and Beta = -0.17 [-0.26,-0.08], t(606) = -4.81, p = <.001, respectively), but not the previous day (p = .566 and p = .31, respectively). The absolute difference between KAP and self-reported total bedtime was not moderated by the relative number of hours with keystroke activity on the previous or following day (p = .377 and p = .604, respectively).

For the difference scores for which (near-)statistically significant variations in the magnitude as a function of the (previous) hours with keyboard activity were found, we performed logistic MLMs to inspect the association between the number of (previous) hours during which keyboard activity was detected with the odds of the keyboard-derived estimate occurring later or being shorter than the corresponding self-reported estimate. Results of these models revealed no statistically significant association between the number of hours during which keyboard activity was detected on the day before the rest period and the odds of the last keystroke occurring later than self-reported sleep onset (see Table S.11). The number of hours with keyboard activity on the next day did significantly relate to the odds of the first keystroke occurring later than the self-reported out-of-bed time. These odds decreased when there were relatively more hours with keyboard activity detected on the next day (with 10% per additional hour with keyboard activity). There was a non-significant trend for a relation with the number of hours during which keyboard activity was detected on the day after the rest period and the odds of KAP being shorter than the self-reported total sleep period. The odds of KAP being shorter than self-reported total sleep period increased by 11% per additional hour of keyboard activity on the following day. The number of hours during which keyboard activity was detected on the day after the rest period was not significantly related to the odds of midpoint KAP occurring after self-reported mid-sleep time or the odds of the first keystroke occurring later than the self-reported sleep offset.

**Table S.11.** Results of logistic multilevel models investigating the direction of the difference scores as a function of (previous) hours with keyboard activity.

| Predictor | Outcome parameter | Estimate | SE | Z | P |
| --- | --- | --- | --- | --- | --- |
| Previous hours with keyboard activity | Last keystroke - sleep onset > 0 | 1.01 | 0.05 | 0.18 | .857 |
| Hours with keyboard activity | Midpoint KAP - mid-sleep time > 0 | 0.98 | 0.04 | -0.53 | .598 |
|  | First keystroke – sleep offset time > 0 | 0.93 | 0.04 | -1.55 | .121 |
|  | First keystroke - out-of-bed time > 0 | 0.90 | 0.04 | -2.91 | .004 |
|  | KAP – total sleep period < 0 | 1.11 | 0.05 | 2.11 | .035 |

*Note.* The parameter estimates represent the exponentiated estimates. The parameter estimates of the difference scores for the timing-related variables refer to the percentage of increase in the odds of the keystroke-derived estimates occurring later than the self-reported estimates. The parameter estimate of the difference between KAP and total sleep period refers to the percentage of increase in the odds of KAP being shorter than the self-reported total sleep time.

## Cross-level Moderations in the Relational Strength between the Keyboard-derived and Self-reported Estimates by Chronotype, General Sleep Quality and Trait Self-control

Tables S.12 to S.19 show the results of the MLMs in which the potential moderating role of chronotype, general sleep quality and trait self-control in the associations between the keyboard-derived and self-reported estimates was investigated.

**Table S.12.** Results of multilevel model for self-reported bedtime with last keystroke, chronotype, general sleep quality and trait self-control as predictors.

| Predictor | Beta | .5% | 99.5% | t | df | p |
| --- | --- | --- | --- | --- | --- | --- |
| Last keystroke - participant mean | 0.26 | 0.15 | 0.36 | 6.25 | 89.8 | <.001 |
| Last keystroke | 0.62 | 0.50 | 0.74 | 12.39 | 72.9 | <.001 |
| Chronotype (MSFsc) | 0.08 | -0.02 | 0.19 | 2.06 | 87.3 | .043 |
| General sleep quality (PSQI score) | -0.02 | -0.11 | 0.07 | -0.49 | 85.5 | .624 |
| Trait self-control | -0.07 | -0.16 | 0.02 | -2.10 | 82.4 | .039 |
| Last keystroke * Chronotype (MSFsc) | 0.08 | -0.04 | 0.20 | 1.66 | 75.2 | .102 |
| Last keystroke * General sleep quality (PSQI) | -0.13 | -0.25 | -0.01 | -2.80 | 75.6 | .007 |
| Last keystroke * Trait self-control | 0.05 | -0.08 | 0.17 | 0.94 | 72.6 | .350 |

**Table S.13.** Results of multilevel model for self-reported try-to-sleep time with last keystroke, chronotype, general sleep quality and trait self-control as predictors.

| Predictor | Beta | .5% | 99.5% | t | df | p |
| --- | --- | --- | --- | --- | --- | --- |
| Last keystroke - participant mean | 0.17 | 0.06 | 0.28 | 3.96 | 88.3 | <.001 |
| Last keystroke | 0.61 | 0.49 | 0.73 | 12.28 | 69.4 | <.001 |
| Chronotype (MSFsc) | 0.17 | 0.06 | 0.28 | 4.11 | 86.6 | <.001 |
| General sleep quality (PSQI score) | 0.05 | -0.04 | 0.15 | 1.46 | 85.9 | .148 |
| Trait self-control | -0.10 | -0.19 | <.01 | -2.59 | 83.1 | .011 |
| Last keystroke * Chronotype (MSFsc) | 0.06 | -0.06 | 0.18 | 1.35 | 71.7 | .182 |
| Last keystroke * General sleep quality (PSQI) | -0.10 | -0.22 | 0.02 | -2.20 | 71.8 | .031 |
| Last keystroke * Trait self-control | 0.03 | -0.10 | 0.15 | 0.53 | 67.8 | .600 |

**Table S.14.** Results of multilevel model for self-reported sleep onset with last keystroke, chronotype, general sleep quality and trait self-control as predictors.

| Predictor | Beta | .5% | 99.5% | t | df | p |
| --- | --- | --- | --- | --- | --- | --- |
| Last keystroke - participant mean | 0.18 | 0.06 | 0.29 | 3.92 | 87.4 | <.001 |
| Last keystroke | 0.62 | 0.50 | 0.74 | 12.33 | 67.9 | <.001 |
| Chronotype (MSFsc) | 0.14 | 0.03 | 0.25 | 3.20 | 85.7 | .002 |
| General sleep quality (PSQI score) | 0.10 | <.01 | 0.20 | 2.68 | 85.2 | .009 |
| Trait self-control | -0.10 | -0.20 | <.01 | -2.52 | 82.6 | .014 |
| Last keystroke * Chronotype (MSFsc) | 0.05 | -0.07 | 0.17 | 1.05 | 70.5 | .296 |
| Last keystroke * General sleep quality (PSQI) | -0.09 | -0.21 | 0.03 | -2.00 | 70.0 | .049 |
| Last keystroke * Trait self-control | 0.01 | -0.11 | 0.14 | 0.26 | 66.5 | .797 |

**Table S.15.** Results of multilevel model for self-reported mid-sleep timing with midpoint KAP, chronotype, general sleep quality and trait self-control as predictors.

| Predictor | Beta | .5% | 99.5% | t | df | p |
| --- | --- | --- | --- | --- | --- | --- |
| Midpoint KAP - participant mean | 0.30 | 0.19 | 0.40 | 7.17 | 86.6 | <.001 |
| Midpoint KAP | 0.61 | 0.54 | 0.69 | 20.06 | 55.3 | <.001 |
| Chronotype (MSFsc) | 0.12 | 0.02 | 0.23 | 3.03 | 88.2 | .003 |
| General sleep quality (PSQI score) | 0.05 | -0.03 | 0.14 | 1.65 | 85.8 | .104 |
| Trait self-control | -0.05 | -0.14 | 0.03 | -1.68 | 84.0 | .097 |
| Midpoint KAP * Chronotype (MSFsc) | 0.04 | -0.04 | 0.11 | 1.31 | 44.7 | .196 |
| Midpoint KAP * General sleep quality (PSQI) | -0.10 | -0.18 | -0.02 | -3.24 | 75.3 | .002 |
| Midpoint KAP * Trait self-control | 0.01 | -0.07 | 0.09 | 0.29 | 58.2 | .773 |

**Table S.16.** Results of multilevel model for self-reported sleep offset with first keystroke, chronotype, general sleep quality and trait self-control as predictors.

| Predictor | Beta | .5% | 99.5% | t | df | p |
| --- | --- | --- | --- | --- | --- | --- |
| First keystroke - participant mean | 0.18 | 0.04 | 0.31 | 3.42 | 91.23 | .001 |
| First keystroke | 0.52 | 0.41 | 0.64 | 11.42 | 78.85 | <.001 |
| Chronotype (MSFsc) | 0.24 | 0.11 | 0.37 | 4.78 | 91.25 | <.001 |
| General sleep quality (PSQI score) | -0.02 | -0.14 | 0.10 | -0.45 | 90.98 | .655 |
| Trait self-control | -0.01 | -0.13 | 0.11 | -0.24 | 88.66 | .814 |
| First keystroke * Chronotype (MSFsc) | 0.08 | -0.04 | 0.19 | 1.67 | 75.56 | .099 |
| First keystroke * General sleep quality (PSQI) | -0.15 | -0.26 | -0.04 | -3.50 | 78.07 | .001 |
| First keystroke * Trait self-control | 0.08 | -0.04 | 0.19 | 1.69 | 92.48 | .094 |

**Table S.17.** Results of multilevel model for self-reported out-of-bed time with first keystroke, chronotype, general sleep quality and trait self-control as predictors.

| Predictor | Beta | .5% | 99.5% | t | df | p |
| --- | --- | --- | --- | --- | --- | --- |
| First keystroke - participant mean | 0.17 | 0.05 | 0.29 | 3.68 | 94.16 | <.001 |
| First keystroke | 0.57 | 0.44 | 0.70 | 11.01 | 80.15 | <.001 |
| Chronotype (MSFsc) | 0.17 | 0.06 | 0.29 | 3.87 | 92.25 | <.001 |
| General sleep quality (PSQI score) | 0.01 | -0.09 | 0.12 | 0.26 | 92.35 | .797 |
| Trait self-control | -0.06 | -0.17 | 0.04 | -1.59 | 90.01 | .115 |
| First keystroke * Chronotype (MSFsc) | 0.06 | -0.07 | 0.19 | 1.14 | 78.51 | .256 |
| First keystroke * General sleep quality (PSQI) | -0.11 | -0.24 | 0.02 | -2.27 | 77.85 | .026 |
| First keystroke * Trait self-control | 0.05 | -0.08 | 0.19 | 1.04 | 92.01 | .303 |

**Table S.18.** Results of multilevel model for self-reported total bed period with KAP, chronotype, general sleep quality and trait self-control as predictors.

| Predictor | Beta | .5% | 99.5% | t | df | p |
| --- | --- | --- | --- | --- | --- | --- |
| KAP - participant mean | 0.13 | <.01 | 0.26 | 2.56 | 87.47 | .012 |
| KAP | 0.54 | 0.40 | 0.68 | 9.64 | 65.60 | <.001 |
| Chronotype (MSFsc) | <.01 | -0.12 | 0.13 | <.01 | 88.94 | .996 |
| General sleep quality (PSQI score) | 0.05 | -0.07 | 0.18 | 1.13 | 87.55 | .262 |
| Trait self-control | 0.04 | -0.08 | 0.17 | 0.89 | 85.16 | .375 |
| KAP * Chronotype (MSFsc) | 0.14 | <.01 | 0.29 | 2.51 | 71.97 | .014 |
| KAP * General sleep quality (PSQI) | -0.09 | -0.22 | 0.04 | -1.76 | 55.78 | .084 |
| KAP * Trait self-control | -0.02 | -0.16 | 0.12 | -0.34 | 73.09 | .738 |

**Table S.19.** Results of multilevel model for self-reported total sleep period with KAP, chronotype, general sleep quality and trait self-control as predictors.

| Predictor | Beta | .5% | 99.5% | t | df | p |
| --- | --- | --- | --- | --- | --- | --- |
| KAP - participant mean | 0.05 | -0.08 | 0.19 | 1.00 | 82.26 | .320 |
| KAP | 0.53 | 0.39 | 0.68 | 9.12 | 69.13 | <.001 |
| Chronotype (MSFsc) | 0.05 | -0.09 | 0.19 | 0.92 | 86.46 | 0.363 |
| General sleep quality (PSQI score) | -0.09 | -0.22 | 0.05 | -1.67 | 84.48 | .100 |
| Trait self-control | 0.11 | -0.02 | 0.25 | 2.15 | 82.46 | .034 |
| KAP * Chronotype (MSFsc) | 0.15 | <.01 | 0.31 | 2.64 | 74.98 | .010 |
| KAP * General sleep quality (PSQI) | -0.09 | -0.23 | 0.05 | -1.66 | 59.59 | .102 |
| KAP * Trait self-control | -0.02 | -0.16 | 0.13 | -0.29 | 77.70 | .773 |

## Cross-level Moderations in the Time Differences between the Keyboard-derived and Self-reported Estimates by Chronotype, General Sleep Quality and Trait Self-control

MLMS with the absolute time differences between the keyboard-derived and the self-reported estimates regressed on chronotype, general sleep quality and trait self-control were run to investigate potential interindividual variations in the magnitude of the time differences between the two assessment modalities as a function of these trait variables related to sleep and sleep habits. The results showed that general sleep quality was the most pronounced predictor for the absolute time differences (see Table S.20). A higher PSQI score (i.e., lower general sleep quality) was associated with larger differences between the last keystroke and the corresponding self-reported estimates (all p < .01). The absolute time lag between the first keystroke and sleep offset was also larger among students reporting more disturbances in their general sleep quality (higher PSQI score). The magnitude of the differences between the midpoint KAP and self-reported mid-sleep time and between the first keystroke and out-of-bed time both showed a non-significant trend for larger differences with a higher PSQI score. KAP showed larger differences with the total bed period and total sleep period among participants with more disturbances in general sleep quality. Chronotype showed no statistically significant association with any of the absolute differences (all p > .05). Trait self-control also showed no statistically significant association with the differences between the keyboard-derived and self-reported estimates (all p > .01), but suggested a non-significant trend for a smaller absolute time difference between the last keystroke and try-to-sleep time, and between midpoint KAP and mid-sleep time among participants with a higher trait self-control (Table S.20).

**Table S.20.** Results MLMs with trait variables as predictors for the time differences between keyboard-derived and self-reported estimates of rest-activity timing and duration.

| Difference score | Predictor | Beta | .5% | 99.5% | t | Df | p |
| --- | --- | --- | --- | --- | --- | --- | --- |
| Last keystroke - bedtime | Chronotype | -0.05 | -0.19 | 0.08 | -1.00 | 88.9 | .319 |
|  | Disturbances in sleep quality (PSQI) | 0.17 | 0.04 | 0.31 | 3.33 | 87.5 | .001 |
|  | Trait self-control | -0.02 | -0.16 | 0.11 | -0.40 | 83.0 | .692 |
| Last keystroke - try-to-sleep time | Chronotype | -0.05 | -0.21 | 0.10 | -0.87 | 90.7 | .387 |
|  | Disturbances in sleep quality (PSQI) | 0.17 | 0.02 | 0.33 | 2.83 | 90.1 | .006 |
|  | Trait self-control | -0.13 | -0.28 | 0.03 | -2.06 | 86.3 | .043 |
| Last keystroke - sleep onset | Chronotype | -0.07 | -0.23 | 0.10 | -1.03 | 89.0 | .307 |
|  | Disturbances in sleep quality (PSQI) | 0.19 | 0.02 | 0.36 | 2.95 | 88.8 | .004 |
|  | Trait self-control | -0.10 | -0.27 | 0.06 | -1.58 | 85.1 | .118 |
| Midpoint KAP - mid-sleep time | Chronotype | -0.03 | -0.16 | 0.09 | -0.68 | 90.9 | .497 |
|  | Disturbances in sleep quality (PSQI) | 0.12 | <.01 | 0.25 | 2.59 | 88.1 | .011 |
|  | Trait self-control | -0.10 | -0.22 | 0.03 | -2.03 | 84.2 | .045 |
| First keystroke - sleep offset | Chronotype | -0.04 | -0.19 | 0.10 | -0.75 | 84.7 | .455 |
|  | Disturbances in sleep quality (PSQI) | 0.17 | 0.03 | 0.32 | 3.14 | 83.5 | .002 |
|  | Trait self-control | -0.06 | -0.20 | 0.08 | -1.10 | 80.8 | .276 |
| First keystroke – out-of-bed time | Chronotype | -0.04 | -0.17 | 0.10 | -0.67 | 84.9 | .502 |
|  | Disturbances in sleep quality (PSQI) | 0.13 | -0.01 | 0.27 | 2.46 | 84.5 | .016 |
|  | Trait self-control | -0.02 | -0.16 | 0.11 | -0.45 | 81.4 | .656 |
| KAP - total bed period | Chronotype | -0.08 | -0.24 | 0.09 | -1.20 | 85.8 | .232 |
|  | Disturbances in sleep quality (PSQI) | 0.22 | 0.06 | 0.39 | 3.56 | 85.0 | .001 |
|  | Trait self-control | -0.10 | -0.27 | 0.06 | -1.62 | 82.4 | .108 |
| KAP - total sleep period | Chronotype | -0.03 | -0.17 | 0.10 | -0.60 | 85.6 | .553 |
|  | Disturbances in sleep quality (PSQI) | 0.16 | 0.03 | 0.29 | 3.10 | 84.2 | .003 |
|  | Trait self-control | -0.04 | -0.18 | 0.09 | -0.84 | 80.6 | .405 |

For the difference scores for which (near-)significantly association with general sleep quality or trait self-control were found, we performed logistic MLMs to inspect the association between these trait variables and the odds of the keyboard-derived estimate occurring later or being shorter than the corresponding self-reported estimate. Results of these models revealed no significant association between the direction of the time differences between the keyboard-derived and self-reported estimates and general sleep quality (Table S.21). Moreover, trait self-control did not significantly predict the direction of the time lag between the timing of the last keystroke and try-to-sleep time or between midpoint KAP and self-reported mid-sleep time (p = .41 and p = .47, respectively).

**Table S.21.** Results of logistic multilevel model for direction of the difference scores as a function of general sleep quality.

| Outcome parameter | Estimate | SE | Z | p |
| --- | --- | --- | --- | --- |
| Last keystroke - bedtime > 0 | 1 | 0.07 | <.01 | .997 |
| Last keystroke - try-to-sleep time > 0 | 1.03 | 0.06 | 0.39 | .696 |
| Last keystroke - sleep onset > 0 | 0.98 | 0.07 | -0.24 | .814 |
| Midpoint KAP - mid-sleep time > 0 | 0.93 | 0.06 | -1.28 | .199 |
| First keystroke - sleep offset > 0 | 0.91 | 0.08 | -1.19 | .236 |
| First keystroke - out-of-bed time > 0 | 0.97 | 0.07 | -0.47 | .641 |
| KAP - total bed period < 0 | 1.05 | 0.08 | 0.59 | .554 |
| KAP - total sleep period < 0 | 1.04 | 0.09 | 0.45 | .649 |

*Note.* The parameter estimates represent the exponentiated estimates. The parameter estimates of the difference scores for the timing-related variables refer to the percentage of increase in the odds of the keystroke-derived estimates occurring later than the self-reported estimates. The parameter estimate of the difference between KAP and total sleep period refers to the percentage of increase in the odds of KAP being shorter than the self-reported total sleep time.

## Relationship between General Sleep Quality and Keyboard Absence Period and the Number of Keyboard Interaction Events during the Self-Reported Total Sleep Period

Keyboard interactions were detected during the self-reported total sleep period on 25.6% of the nights. Figure S.4 displays the number of keyboard interaction events that were registered during the self-reported total sleep period per frequency bin. A logistic MLM was performed to inspect the association between general sleep quality and the likelihood that at least one keystroke was registered during the self-reported total sleep period. Results revealed that the occurrence of keyboard interaction events during the self-reported total sleep period was not statistically significant associated with participants’ PSQI score (Estimate = 1.08, SE = .08, Z = .99, p = .32). Moreover, a MLM inspecting the relation between general sleep quality and KAP showed that the size of KAP was not significantly associated with participants’ PSQI scores (B = .02, SE = .05, t(97) = .36, p = .72).

**Figure S.4.** Histogram of the number of keyboard interaction events during the self-reported total sleep period.


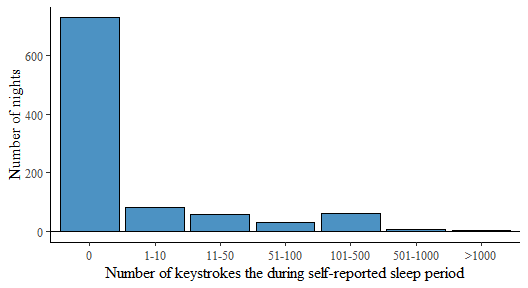

Supplement: Multimedia Appendix 1 [file jmir_v25i1e38066_app1.docx]
